# Crossmodal semantic congruence and rarity improve episodic memory

**DOI:** 10.3758/s13421-024-01659-9

**Published:** 2025-02-19

**Authors:** Pau Alexander Packard, Salvador Soto-Faraco

**Affiliations:** 1 Center for Brain and Cognition, Universitat Pompeu Fabra, Carrer de Ramon Trias Fargas, 25-27, 08005 Barcelona, Spain; 2 Institució Catalana de Recerca I Estudis Avançats, ICREA, Barcelona, Spain

**Keywords:** Episodic memory, Incidental memory, Recollection, Salience, Crossmodal, Ageing

## Abstract

Semantic congruence across sensory modalities at encoding of information has been shown to improve memory performance over a short time span. However, the beneficial effect of crossmodal congruence is less well established when it comes to episodic memories over longer retention periods. This gap in knowledge is particularly wide for cross-modal semantic congruence under incidental encoding conditions, a process that is especially relevant in everyday life. Here, we present the results of a series of four experiments (total *N* = 232) using the dual-process signal detection model to examine crossmodal semantic effects on recollection and familiarity. In Experiment 1, we established the beneficial effects of crossmodal semantics in younger adults: hearing congruent compared with incongruent object sounds during the incidental encoding of object images increased recollection and familiarity after 48 h. In Experiment 2 we reproduced and extended the finding to a sample of older participants (50–65 years old): older people displayed a commensurable crossmodal congruence effect, despite a selective decline in recollection compared with younger adults. In Experiment 3, we showed that crossmodal facilitation is resilient to large imbalances between the frequency of congruent versus incongruent events (from 10 to 90%): Albeit rare events are more memorable than frequent ones overall, the impact of this rarity effect on the crossmodal benefit was small, and only affected familiarity. Collectively, these findings reveal a robust crossmodal semantic congruence effect for incidentally encoded visual stimuli over a long retention span, bearing the hallmarks of episodic memory enhancement.

**Supplementary Information:**

The online version contains supplementary material available at 10.3758/s13421-024-01659-9.

## Introduction

Someone attending a symphony concert can recognize some of the musical instruments of the orchestra by sight, and in some cases, match their characteristic sounds by ear. This process requires access to crossmodal associations acquired during a lifetime regarding which sounds correspond to which visual objects. Generally speaking, it would seem rather advantageous for humans to harness on this kind of crossmodal relations from semantic memory, both to expedite the perception of events in the immediate sensory environment (as it has been shown in multiple studies on crossmodal perception and attention; see Beauchamp et al., 2004; Chen & Spence, 2018; Doehrmann and Naumer, 2008; Iordanescu et al., 2008; Kvasova et al., 2019, 2021, 2023; Laurienti et al., 2004; Mastroberardino et al., 2015; Molholm et al., 2004; for reviews see, Shams & Seitz, 2008; Stein, 2011), as well as to form and retain richer memories of these events for later retrieval. Indeed, a substantial number of studies have reported increased memory for events originally encoded along with semantically corresponding information in more than one sensory modality (see Matusz et al., 2017; Murray & Shams, 2023; Soto-Faraco & Spence, 2025; Thelen & Murray, 2013, for reviews). However, despite the growing number of such multisensory memory studies, some important questions remain unanswered.

One particularly relevant question is to understand if and how implicit encoding of semantically congruent multisensory events improves episodic memory—that is to say, memories about crossmodal events experienced without an intentional, explicit effort made to register and retain them. Although the effects of crossmodal semantic congruence after incidental encoding have been addressed profusely, studies have almost exclusively used short-term memory protocols, and evidence regarding episodic long-term memory is scarce. Particularly lacking is evidence regarding long retention intervals, in the order of hours and days. This is precisely the main question we address here. To approach this question, we have adopted the dual-process theory framework (e.g., Koen et al., 2017; Yonelinas 2002; 2010), which separates the contribution of familiarity and recollection processes and can therefore provide valuable information regarding the nature of potential memory improvements. In addition to the central question regarding the possible benefits of crossmodal semantic congruence over long intervals, our study has taken us to investigate two further related issues which have not received sufficient attention in prior crossmodal memory literature. On the one hand, whether the effects of crossmodal congruence on memory vary at an older age, as this may have important practical implications (Heikkilä et al., 2018). On the other hand, whether crossmodal congruence effects are modulated by relative frequency, or rarity, of semantically in/congruent events. Despite the relative frequency of in/congruent events has been pointed out as a potential source of confound in previous studies (Pecher & Zeelenberg, 2022; Thelen et al., 2015), this variable has rarely ever been investigated systematically. Below, we introduce the relevance of these questions in more detail and describe the approach and scope of the present study.

### The hypothesis for crossmodal memory benefits: Context effects and redintegration

The hypothesis that crossmodal semantic congruence at encoding will benefit later retrieval can be linked to improvements due to context congruence, extensively reported in memory literature. For example, memory for prose improves following the preactivation of related information (Bransford & Johnson, 1972), as well as memory for items presented within a context that is semantically compatible, rather than incompatible (Bein et al., 2015; Packard et al., 2017, 2020; Schulman, 1974; van Kesteren et al., 2012). One accepted explanation of these context-congruence effects is that the successful integration of a new event within a coherent context increases the elaboration or ‘spread′ of the encoding, as compared with new events occurring in a semantically incongruent context (Bein et al., 2015; Craik & Tulving, 1975). The hypothesis that, more or less explicitly stated, underlies many previous multisensory memory studies is that semantic congruence across modalities may produce similar context-beneficial effects, causing increased spread of information encoding and enhancing later recognition. This is the basis of various current accounts of crossmodal memory gains.

For example, various crossmodal studies addressed the idea that when the different single-modality components of a crossmodal event are encoded as a unitary coherent representation, the whole multisensory representation can be later retrieved upon reencounter with just one of its original components. This hypothesis, based on Hamilton’s principle of *redintegration* (see Hollingworth, 1920), was tested by Nyberg et al. (2000) in a PET study. Nyberg et al. found that the recognition of images that had been previously encoded along with their semantically corresponding sounds produced stronger activity in the auditory cortex compared with reencounter with images that had been previously encoded under single-modality conditions (see Wheeler et al., 2000, for a similar result using fMRI). These findings suggested that multisensory encoding leads to richer episodic memories, compared with those of single modality events. Despite the original study by Nyberg et al. (2000) did not find behavioural evidence for such crossmodal advantage (and Wheeler et al., 2000, did not test it), a growing number of studies since then have provided positive evidence. Most of these later studies support the multisensory memory reactivation account (see Matusz et al., 2017; Thelen et al., 2012), which propose that crossmodal congruent events form richer memories based on multisensory representations. We review them next.

### Crossmodal memory gains in continuous recognition

Evidence showing memory improvements for semantically congruent audiovisual stimuli has been tested, using relatively short retention intervals, with the continuous recognition task (e.g., Lehmann & Murray, 2005; M. M. Murray et al., 2004, 2005; Thelen et al., 2015). In this task, participants are asked to detect repeated items in a serial presentation stream of object images (line drawings), with repetitions occurring every four to 21 items (e.g., retention intervals of approximately, 8–42 s). These reports are often not explicit about which memory system is tapped into with this task, though it has been described as a working memory task (Thelen et al., 2012) and an episodic memory task (Matusz et al., 2017). The key experimental manipulation is whether the initial presentation of an object image is unisensory, it is accompanied by the characteristic sound of the depicted object, or, in some of the studies, accompanied by a semantically incongruent sound (e.g., Lehmann & Murray, 2005; Thelen et al., 2015). Their results have almost invariably showed a memory advantage for images initially encountered with crossmodally congruent sounds (improvements ranging from 2 to 8%), both in recognition percentage as well as when using signal detection measures such as *d′*. Similar effects have also been consistently reported for sound memory when encoded along with congruent images (Matusz et al., 2015; Moran et al., 2013; Radecke et al., 2022; Thelen et al., 2015). The continuous recognition task provides the strongest evidence to date for crossmodal congruence benefits in incidental memory (though see Pecher & Zeelenberg, 2022, for criticism). However, this task has never been applied to memories beyond just a few seconds old, leaving the question of crossmodal memory benefits over long-term episodic memories unanswered. One of the main purposes of the present study is to ascertain if crossmodal congruence benefits extend over longer maintenance periods and display some of the hallmarks of episodic memory.

### Evidence for crossmodal effects in episodic memory

Other studies have addressed crossmodal semantic congruence effects in episodic memory, using relatively longer retention periods, larger memory loads, and sometimes intervening distractor tasks to prevent rehearsal. In a seminal study, Thompson and Paivio (1994) used a recall task under explicit as well as incidental study conditions, to show that crossmodally congruent stimuli were better remembered than unisensory ones. However, the authors convincingly showed that this crossmodal facilitation was best explained with a probability summation model. This result, consistent with a dual-coding account, implies that independent memory processes in each modality are enough to explain the observed crossmodal congruence gain, making it unnecessary to postulate a specific role of multisensory integration processes. Interestingly, despite the profound implications of this conclusion, it has hardly ever been tested in the subsequent 30 years (though see Meyerhoff et al., 2023, who reached a similar conclusion). As part of a series of studies on crossmodal memory, Heikkilä et al. (2015) and Heikkilä and Tiippana (2016) tested whether the visual component of a semantically congruent auditory-visual event was better remembered after an intentional study phase, than the visual component of an auditory-visual event with meaningless (e.g., a picture of a dog with a burst of noise) or semantically incongruent sounds (e.g., a picture of a dog with a guitar chord sound). In their experiments, participants were asked explicitly to study lists of about 52 items, and then perform an old/new recognition task immediately after. The results were mixed, as one study with adults failed to find any crossmodal congruence improvements (Heikkilä et al., 2015) and another study, with children, reported a crossmodal congruence gain for images (Heikkilä & Tiippana, 2016). Heikkilä et al. also addressed crossmodal congruence gains for sound memory in several other studies (Heikkilä & Tiippana, 2016; Heikkilä et al., 2017, 2018), this time with consistently positive outcomes.

It is worth noting that Heikkilä et al. (2018) is the only study we know of that has compared crossmodal congruence gains between young and older adults (mean age 23 vs. 71 years old, respectively). In this study, Heikkilä et al. tested memory for object sounds and found that younger adults benefited relatively more than older adults from crossmodal congruence at encoding (though this pattern reversed when memory for spoken words was tested). Heikkilä et al. attributed the stronger crossmodal gain in younger adults to poorer auditory and visual encoding in the older adults, given the short duration of events at encoding in their experiment (400 ms). In the present study, we test crossmodal memory gains across age groups using longer stimulus presentations (4 s), possibly preventing the potential differences in encoding at a perceptual stage. In addition, thanks to the dual-process signal detection (DPSD) approach, to be described in more detail below, in the present study it will be possible to discern group differences owing to perceptual encoding efficiency, from those related to better episodic representations.

In all their experiments, Heikkilä et al. used only intentional, explicit study protocols. Studying incidental memory may be of particular interest, however, since it is more prevalent in everyday life routines than intentional memory, and it tends to decline more rapidly with age (Eysenck, 1974; Kontaxopoulou et al., 2017; Lair et al., 1969). Therefore, finding out how possible crossmodal beneficial effects play out in long-term memory after incidental encoding is of marked interest to understand potential practical implications for mnemonic training and rehabilitation (Cicerone et al., 2019; Craik et al., 2007). According to some authors, incidental memory protocols have the additional advantage of avoiding the effects of possible individual differences in active encoding or rehearsal strategies which might conflate with memory performance per se (Kontaxopoulou et al., 2017; Vingerhoets et al., 2005).

### Span and nature of crossmodal congruence benefits

Most of the studies discussed so far have used recognition (old/new) or, to a lesser extent, recall tasks focusing on the identity of the to-be-remembered object. Recent studies have reported positive effects of crossmodal congruence also for location memory (Marian et al., 2021), suggesting that episodes render richer memory representations when encoded as part of a crossmodally congruent event. Of particular interest here, Duarte et al. (2023) used the dual-process theory model (e.g., Koen et al., 2017; Yonelinas 2002, 2010) to reveal that the memory gain for crossmodally congruent events was strongly based on recollection, instead of familiarity processes. Duarte et al. concluded that crossmodal semantic congruence led to a qualitatively different episodic encoding of the event, with richer representation of specific details. However, like most of the episodic memory studies discussed so far, both Marian et al. (2021) and Duarte et al. used relatively short retention intervals, in the order of minutes. Despite their results being informative to understand episodic memory of crossmodal events, they remain inconclusive with respect to the effect of multisensory congruence when it comes to more substantial retention intervals. Examining these effects on longer retention intervals that span hours or days is necessary to test the generality of audiovisual congruence effects. According to well accepted two-stage memory models, longer retention intervals may involve qualitatively different processes to act upon the stored memories, especially when sleep periods span between encoding and retrieval (e.g., Born & Wilhelm, 2012; Borota et al., 2014; McKenzie & Eichenbaum, 2011). The only studies to test for crossmodal congruence effects over long retention intervals, Meyerhoff and Huff (2016) and Meyerhoff et al. (2023), used short movie clips with either matching or mismatching video and soundtracks. In both studies, the authors found better memory for matching film fragments after 1 day, and in Meyerhoff and Huff even after 1 week. The materials used in Meyerhoff et al. are, however, hard to compare with the object image–sound pairings typically used in all other studies, where semantic manipulations involve congruence or incongruence, for single well-identifiable objects, at the basic semantic category level. The cross-spliced film fragments used by Meyerhoff et al.’s manipulated crossmodal *semantic* congruence at a more general level, also possibly including (in)congruence at lower, sensory levels.

### Recollection versus familiarity in long-term memory

According to the dual-process theory, performance on long-term memory recognition tests has been proposed to depend on two main types of retrieval processes, that rely on different networks of brain regions: recollection whereby participants retrieve qualitative information about an event, and familiarity whereby participants simply have a feeling that they have seen or heard something before not necessarily accompanied by recollection of more specific details of the context (Diana et al., 2007; Eichenbaum et al., 2007; Yonelinas, 2002; Yonelinas et al., 2005, 2010). Recollection, considered the hallmark of episodic memory (Renoult et al., 2019; Tulving, 2002), is defined as the process, possibly unique to human beings, which allows them to consciously reexperience past situations, to retrieve the related information such as spatial arrangement. Recollection is entwined with a sense of self, autonoetic awareness, and subjective time. The recollection process is especially impaired in amnesic patients (Huppert & Piercy, 1978; Yonelinas, 2002) and older people (Koen & Yonelinas, 2016), and is important for high-confidence memories (Tulving, 2002; Yonelinas, 2002). Receiver operating characteristics (ROC) analysis has helped characterizing and disentangling the contributions of recollection and familiarity, thus furthering our understanding of episodic memory (Koen et al., 2017; Yonelinas, 1994, 2001).

Therefore, one important question is whether memory benefits based on crossmodal semantic congruence could potentially affect recollection, familiarity, or both. Measuring these two different aspects of long-term memory separately is relevant on several counts. First, it can help disentangle a dual-coding account of crossmodal memory gains (Meyerhoff et al., 2023; Thompson & Paivio, 1994), which states that crossmodal effects simply arise from statistical summation of information, from other accounts positing that multisensory encoding renders qualitatively different episodic representations, such as the multisensory memory reactivation account (Matusz et al., 2017). In addition, telling apart recollection from familiarity can provide a test for Heikkilä et al.’s (2018) encoding efficiency account of the reduced crossmodal gain in older individuals, compared with younger ones.

Apart from the recent study by Duarte et al. (2023), we could find no previous studies on how recollection and familiarity differentially contribute to crossmodally encoded memories. By analogy to levels of processing studies using an encoding protocol comparable to the one used here, we might expect an increase in recollection (Voss & Paller, 2017), but also a smaller yet consistent increase in familiarity (Yonelinas, 2002) for semantically congruent audiovisual stimuli. Studies using the levels of processing framework have found that processing the meaning of stimuli during encoding, as opposed to processing tasks that concern only the perceptual components of the stimuli, increases both recollection and familiarity (Yonelinas, 2002). More specifically, recollection often proves to be more sensitive to effects after deeper encoding than familiarity (Yonelinas, 2002). If evaluating crossmodal semantic congruence during encoding involves similar meaning processing as in the levels of processing paradigms, we can expect similar outcomes on subsequent memory. Duarte et al. have found some support for this using an incidental encoding task that required a relatively deep level of processing (the task involved judging ‘can this object fit in a suitcase?’) and a surprise memory test afterwards. Their results showed crossmodal gain in recollection but not in familiarity, when comparing memory for images encoded as part of crossmodally congruent events with controls (images encoded either with a meaningless noise or with an incongruent sound).

### Scope of the present study

The main goal this study was to address the impact of crossmodal congruence on episodic memory over long retention intervals, for images that are encoded under incidental situations. This basic question is addressed in four experiments. The main approach of Experiments 1 and 2 is based on the DPSD (e.g., Koen et al., 2017; Yonelinas, 2002; 2010), with the aim to first establish the principle finding and, second, to extrapolate this finding across different age groups. Given the expected general decline in memory performance for the older group, we deemed it important to address potential age differences in the strength the crossmodal benefit. So far, only one study has addressed crossmodal memory effects in an elderly sample (Heikkilä et al., 2018). The present study addressed the question of frequency distribution, or rarity, of crossmodal incongruence, in Experiment 3. The distribution of semantically incongruent events throughout the experimental materials is interesting in the light of known effects of rarity and oddity in memory. Whereas typical crossmodal studies such as the ones discussed earlier, as well as our Experiments 1 and 2, include congruent and incongruent events in approximately equal proportion, one might argue that blatant semantic incongruences are rare in real life, and if we encounter them, they tend to be quite memorable. At least one earlier study (Glicksohn et al., 2023) found superior performance for rare, highly salient, incongruent crossmodal events, compared with frequent semantically congruent ones. So, with frequency manipulations in Experiment 3, we tried to separate the effect of rarity and oddity (we hardly ever encounter cats that bark, or violins that sound like a trumpet in real life) from the effect crossmodal (in)congruence per se.

To address these questions, we designed an incidental encoding paradigm in which participants were asked to rate the congruence of the audio and visual components of a list of audiovisual stimuli, without knowing they would be given a memory test at a later moment. Pause et al.’s (2013) criteria were followed to increase ecological validity and reliability of our episodic memory test, including precise timing of encoding and retrieval trials, an incidental memory paradigm in which participants were presented with a delayed (approximately by 48 h) recognition test, and with each stimulus being presented only once during the encoding phase. Furthermore, to assess whether the effects could be associated to recollection retrieval processes, a ROC curve analysis was implemented (Juola et al., 2019; Yonelinas, 2002), which allows calculating the recollection parameter, as defined in the DPSD model (Yonelinas, 2002; Yonelinas et al., 2010). This approach allowed us to estimate two different aspects of memory, recognition and familiarity, for crossmodally congruent and incongruent events. Disentangling between the different processes contributing to a putative crossmodal benefit in memory can be informative as to whether the nature of this effect is due to redundant information, or else involves a distinct effect at a representational level.

The methods, sample size estimation, hypothesis, analysis pipeline, and predictions of experiments were preregistered prior to data processing and analyses. A general description of the project and the pilot experiment can be found online (https://osf.io/9ghvn), and the specific preregistration documents for each experiment can be found in individual files within the associated project (https://osf.io/ja8zu). The Result sections report the planned analyses first, with further exploratory analyses identified as such, after the planned analyses. A pilot experiment was run prior to submitting the initial preregistration to help adjust some of the parameters of the experiment including the final sample size (the results of this pilot experiment are reported in Appendix 1,^1^). The experimental paradigm was run online on the Pavlovia platform (https://pavlovia.org), with custom scripts written in JavaScript with PsychoPy3 (Version 2020.2.10) and edited afterwards (Peirce et al., 2019). The Pavlovia online experiment project, including the research materials used for Session 1, can be accessed publicly here, together with all the data: (https://gitlab.pavlovia.org/Pau/audio_visual_congruence_session_1). The Pavlovia online experiment project used for Session 2 can be accessed publicly here: (https://gitlab.pavlovia.org/Pau/audio_visual_congruence_session2). Across the whole project, 255 of the 287 participants who had completed the encoding phase (Session 1) returned to take the memory test (Session 2)—around an 89% return rate.

This study was approved by the Institutional Committee for Ethical Review of Projects (CIREP) at the Universitat Pompeu Fabra, date 11.02.2021, ref number: 173.

## Experiment 1: Establishing episodic memory effects of crossmodal semantic congruence over a long retention interval

The hypothesis addressed in Experiment 1 is that semantically congruent information increases the elaboration or *spread* of the encoding (i.e., total breadth of analysis carried out), compared with semantically incongruent information, offering a cognitive explanation for the levels of processing effect and the related congruence effect, in line with Craik & Tulving (1975). According to this hypothesis, recollection should be enhanced to the extent that the (crossmodal) information forms an integrated semantically meaningful unit. Therefore, one would expect that semantically congruent multisensory events will be better recollected than incongruent ones because a more elaborate trace is encoded, and because in such cases the structure of semantic memory can be utilized more effectively. What is more, this effect is expected to persist over retention intervals in the order of hours/days, far beyond the typical ones used so far, in the order of minutes. To test this, we measured episodic memory for images of objects that had been presented 2 days prior, either paired with their semantically congruent sounds or paired with semantically incongruent sounds. We use the most widely accepted definition of episodic memory as long-term memory supported by recollection of past events. That is, in accord with the widely accepted dual process view, although recognition may be supported by both recollection and familiarity, conscious recollection is the critical process supporting episodic memory (for reviews, see Tulving, 2002; Yonelinas, 2001), which will be the critical test comparing the congruent and the incongruent condition here.

### Methods

#### Sample-size calculation

Because we were interested in at least a medium effect size on recollection (*d*_z_ = 0.4), using G*Power software (Faul et al., 2007) we calculated that we needed at least 58 participants to assess the medium effect size (*d*_z_ > 0.4) adequately with a one-tailed Wilcoxon signed-rank test (congruent audiovisual stimuli vs. incongruent audiovisual stimuli), with a power of 90% and alpha level of 0.05. We also measured familiarity (F component), and *d′* [*z*(hit rate) − *z*(false-alarm rate)], but the power calculation did not ensure correct sensitivity for additional testing for differences between conditions for F and d′ with Bonferroni corrections for multiple comparisons.

#### Participants

Participants were recruited online through the Prolific platform (http://profilic.com). They were paid at a rate of 7.50 GPB per hour. The final sample was *N* = 58 (29 women). We included only participants between 18 and 35 years of age (mean = 23.78 years, *SD* = 4.21). All participants were fluent in English, declared no hearing or language impairments, no current mental illness that could impact their performance, and possessed normal or corrected-to-normal vision. Based on our pilot data (see below) and previous paradigms using ROC analysis to estimate recollection and familiarity, we set up performance criteria for inclusion of participants’ data: (1) *d′* signal detection measure [*z*(hit rate) − z(false-alarm rate)] over 1 overall, (2) performance on the arithmetic distraction task over 75%, (3) at least 10% of the responses in the intermediate bins (2–5) in the memory test to ensure a proper ROC curve analysis, (4) at least 70% accuracy during encoding task—in other words, minimum 70% agreement of participants’ congruence ratings with our predetermined categories. Nine people were thus excluded in the process of reaching the final sample of 58 included participants: six for criterion (1), 1 for criterion (2), 2 for criterion (3), and 0 for criterion (4).

#### Stimuli and procedure

Three hundred seventy-two (372) image stimuli were selected from the THINGS database (Hebart et al., 2019), including animals, musical instruments, and other easily recognizable objects, for which there is a characteristic sound (e.g., a guitar, a cat, a car), and 372 corresponding sounds were selected from the Freesound website (https://freesound.org/). For the congruent condition, pairings of matching images and sounds were presented together. All images were 330 × 330 pixels in size, with resolution 300 dpi. All sounds were normalized with Audacity software (Version 3.0.0) to a maximum amplitude of − 1 dB relative to full scale or maximum possible digital level (dBFS). For the incongruent condition, images and sounds were selected from different semantic categories (animal, musical, transportation, other objects) and paired together. Participants ran two sessions—one comprising the encoding and the distraction phases and another session comprising the recognition phase, with the later session running about 2 days after the first. Before each session’s task, the protocol included a visual calibration stage: Participants adjusted the size of a rectangle on the screen to the size of a standard credit card (by placing it on top of the drawn rectangle). This allowed us to scale the task elements to the individual screen resolution of each participant’s monitor.

During the encoding phase, 93 congruent and 93 incongruent audiovisual pairings were presented, in random order. Assignment of congruent and incongruent pairings of items was counterbalanced across participants. The onset of the images (4-s duration) and the corresponding sounds (2.5-s duration) were aligned. Participants were instructed to rate the congruence between image and sound on a scale from 1 to 4 (1 = *doesn’t match at all*; 2 = *doesn’t match*; 3 = *matches*; 4 = *matches very well*) within the duration of the trial (from the onset to the offset of the image). Based on previous deep encoding paradigms, these instructions encouraged processing both modalities to judge how well they fit together semantically. Stimuli during encoding were shown for their full duration independently of when the participant responded, to avoid encoding differences associated to variable response times. The interstimulus interval was 1 s. Immediately prior to the beginning of the encoding session, participants ran eight training encoding trials with immediate feedback to indicate whether the response was in time or not, together with reminders of the instructions. They were also reminded to adjust volume to a comfortable level that allowed them to hear the sounds played well and clear. A brief pause with another reminder of the instructions was given halfway through the encoding phase. This phase lasted approximately 25 min.

In the distraction phase, which was run immediately after the encoding phase, participants performed a mathematical task aimed at eliminating possible rehearsal or maintenance strategies that could affect the subsequent recognition memory test differently across participants. In this task, participants were shown pairs of numbers of up to seven digits, side by side. In each trial, they were instructed to respond with the left or right arrow keys as to which of the two numbers was larger. After each trial, feedback was presented indicating whether the participant was correct or not. There was a total of 100 trials, and this phase lasted approximately 2–3 min, including the time to read the instructions and the initial training trials. There were three previous training trials for this task with a reminder of the instruction for the task.

In the test phase, we presented the participants with a recognition memory task. This was done on the second day after the encoding phase, with a retention delay of approximately 48 h. All the 372 image stimuli were presented in a random order without the associated sound. This included all the images presented during the encoding phase, mixed randomly with an equal amount of new but equivalent images. Participants were instructed to respond on a confidence scale from 1 to 6, whether they thought the image had appeared during the initial encoding phase or not: 1 = *sure new*, 2 = *maybe new*, 3 = *guess new*, 4 = *guess old*, 5 = *maybe old*, 6 = *sure old*. Images remained on the screen until the participant responded, for a maximum of 4 s. This test phase started with 16 training trials (not included in analysis) with immediate feedback, presented to indicate whether the participant responded in time or not, together with reminders of the instructions. A brief pause with another reminder of the instructions was given halfway through the test phase. This phase lasted approximately 25 min.

#### Data analyses and modelling

Although we designed the experiment with a priori half congruent and half incongruent items as per the experimenter’s criteria, for the critical memory test comparing memory for congruent versus incongruent items, we considered only those items whose congruence had been judged accordingly by the participant’s individual ratings. Congruent items were items that were initially selected as congruent by design and that the participants rated as 3 or 4 on the 4-point scale, and incongruent items were items that were considered incongruent by design and rated 1 or 2. From here on, when we refer to congruent items, we mean items in the congruent stimulus category that had also been rated as congruent by subjects, and likewise for incongruent items. Please note that this renders a different number of trials per condition in each subject (please see Appendix 4 for descriptive statistics on trial numbers).

Data analyses followed the DPSD (Yonelinas, 2002; Yonelinas et al., 2010), which defines familiarity as a function of the distance between the means of the old and new item distribution and the response criterion *c*, similar to other signal detection models such as the equal-variance signal detection model (EVSD) with a single *d′* parameter (Koen et al., 2014). In contrast, in the hybrid DPSD model, recollection has a retrieval threshold and is considered to make an independent contribution, leading to high confidence responses. A minimum of two functionally independent memory components are necessary to account for recognition performance with dissociated ROC sensitivity and asymmetry (Yonelinas & Parks, 2007), which in the DPSD model are recollection I and familiarity (F). For the DPSD model fitting, lures (new items in the memory test) were not classified as congruent or incongruent and were considered both, as recommended in the ROC toolbox manual (Koen et al., 2017; please see Appendix 5 for a formal definition of model parameters and Appendix 6 for group-wise ROC curves and average parameter fits for each experiment). Besides the DPSD model parameters R and F, we also report the analyses using *d′* throughout, given that this is a measure directly comparable to many previous crossmodal memory studies. For the *d′* calculation, new items were assigned a dummy congruence label randomly and equiprobably for the calculation of false-alarm rates in each condition independently.

The prediction for Experiment 1 was a greater recollection (R parameter) for semantically congruent events compared with semantically incongruent ones. Familiarity and *d′* were included in further exploratory analyses. We used a one-tailed Wilcoxon signed-rank test to evaluate the hypothesis that performance after congruent encoding was superior than after encoding in the incongruent condition. We also included two-sided *t* tests, which are more adequate when the data are normally distributed, to evaluate the exploratory tests more fairly. We used Shapiro–Wilk test for normality.

### Results

#### Encoding and distraction task

Across all items, the average interparticipant congruence rating was 2.24 (*SD* = 0.12) in the 1–4 scale. Participants gave a mean of 43.4% congruent responses (responses 3 or 4 for the congruence rating task, *SD* = 4.06%), and 55.9% incongruent responses (responses 1 or 2, *SD* = 4.06%). Participants gave on average 84.6% (*SD* = 7.2%) congruent responses to audiovisual pairings categorized as congruent by design, hence rendering on average 79.5 (min–max: 60–91) valid items per participant for the congruent condition in the memory test. For pairs categorized as incongruent by design, participants gave on average 96.9% (*SD* = 4.1%) incongruent responses, rendering on average 90.1 (min–max: 72–93) valid items per participant for the incongruent condition in the memory test (see Appendix 4 for distribution of responses). The mean reaction time for congruent responses was 2.21 s (*SD* = 0.58), and for incongruent responses was 2.10 s (*SD* = 0.63). The intraclass correlation coefficient measuring agreement between single fixed raters for the items in the congruence rating task was 0.86. The interparticipant average hit rate for the distraction task right after the encoding phase, where participants had to choose the larger of two numbers, was 0.89 (*SD* = 0.03).

#### Recognition task 

##### DPSD model parameters

**Fig. 1:**
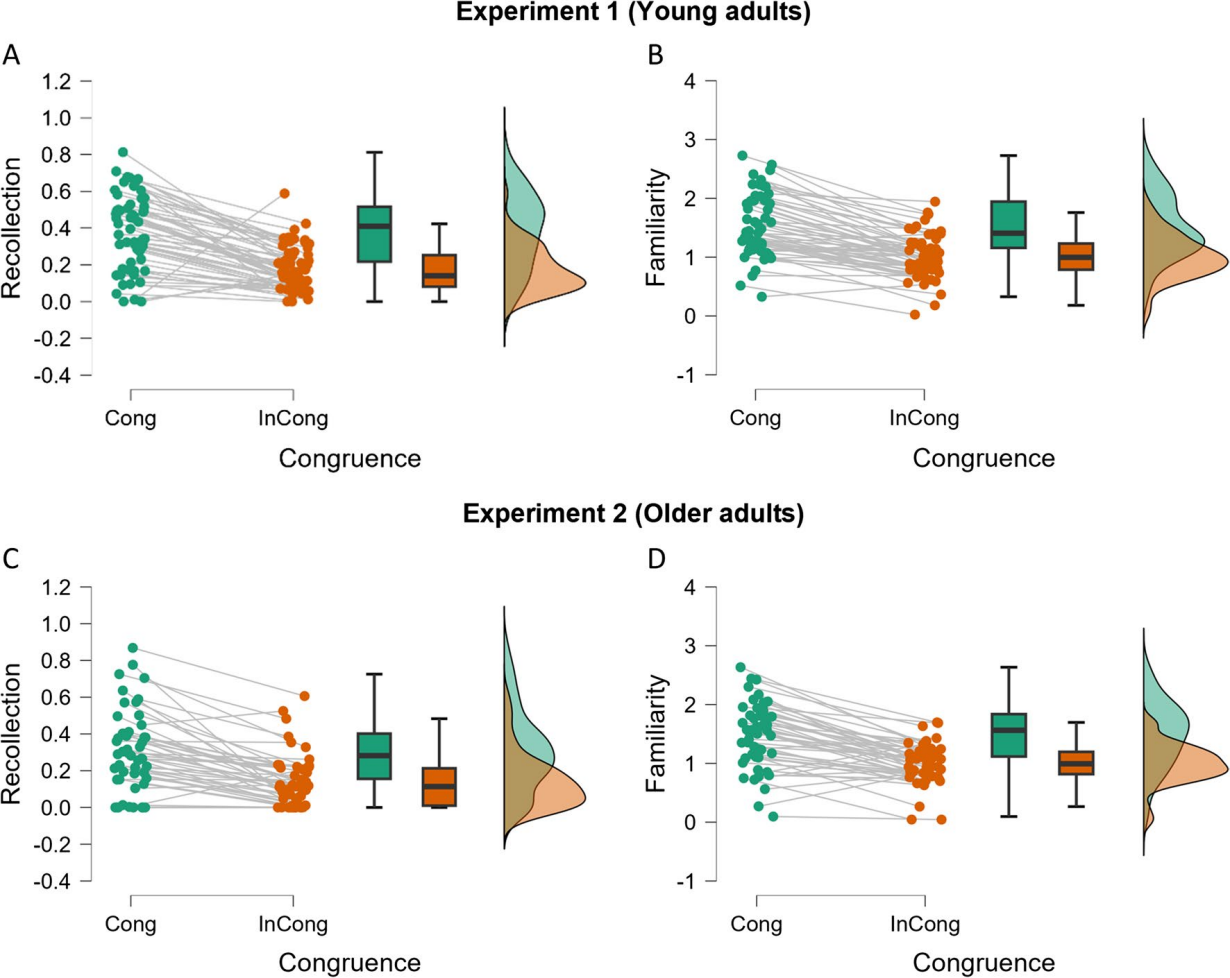
Distribution of individual estimates of the recollection parameter (**A.** and **C.**) and the familiarity parameter (**B.** and **D.**) for each congruence condition in Experiment 1 (young adults; top) and in Experiment 2 (older adults, bottom). (Colour figure online)

Our main analysis in this experiment focused on recollection. Applying the DPSD model, the mean recollection (R parameter; see Fig. 1A) for items rated as congruent, in the congruent condition, was 0.38 (*SD* = 0.20), whereas for items rated as incongruent, in the incongruent condition, was 0.17 (*SD* = 0.12). As predicted, recollection for congruent items was significantly greater than recollection for crossmodally incongruent items (Wilcoxon signed-rank test, one-tailed, *Z* = 5.95, *p* < 0.001). Data deviated from normality (*W* = 0.909, *p* < 0.001). The difference was also significant with a two-tailed* t* test, *t*(57) = 8.60, *p* = 6.89e-12. Cohen’s *d* effect size was 1.13—a large effect size. Regarding the familiarity parameter F (Fig. 1B), for congruent items mean = 1.52 (*SD* = 0.53) was higher compared with incongruent ones mean = 1.00 (*SD* = 0.37). The one-tailed Wilcoxon signed-rank test was significant (*Z* = 6.21, *p* < 0.001). The data were normally distributed (*W* = . 0.983, *p* = 0.587). This difference was also significant with a two-tailed *t* test, *t*(57) = 10.30, *p* = 1.09e-14, Cohen’s* d* = 1.36, indicating a large effect size.

##### *D′* analyses

Like the DPSD parameters, sensitivity to discern old items from new ones was significantly higher for events encoded as part of a congruent multisensory stimulus (Wilcoxon signed-rank test; *Z* = 6.21, *p* < 0.001). The data were normally distributed (*W* = 0.983,* p* = 0.586), and the difference was also significant with a two-tailed *t* test, *t*(57) = 13.05, *p* < 0.001, Cohen’s *d* = 1.812. Complementary analysis showed *d′* scaled with congruence rating at the encoding phase (see Appendix 2), and that the congruence effect applied across the board when including semantic category as a factor (see Appendix 3). These results allow to gain confidence for the use of these materials and protocol in subsequent experiments.

### Discussion

The results to emerge from Experiment 1 are clear. A sample of 58 individuals were presented a set of 186 crossmodal stimuli, during a congruence rating task, of which approximately half the events were semantically congruent across modalities and the other half were incongruent. This ensured incidental encoding, with deep processing of semantic features. The same individuals received an unanticipated recognition memory test approximately 48 h later, where they had to tell items encountered in the encoding phase from amongst a set of 372 items including the 186 old items (half originally associated with congruent sounds and half with incongruent sounds) and 186 new, comparable lures, using a 6-point confidence rating (from *very sure old* to *very sure new*). This protocol allowed us to use a DPSD model to estimate separate recollection and familiarity parameters, atop of the more traditional *d′* parameter from the signal detection theory. In all three measures, we observed large effects of semantic congruence. This means that the subsequent memory effect, encapsulated in the superior sensitivity (*d′*) for events encoded in a crossmodally congruent semantic context, was supported by a more robust memory representation of these items rendering higher recollection, as well as a higher sense of overall familiarity.

These results are in line with other studies that have reported gains related to crossmodal semantic congruence at encoding, compared with incongruence (Matusz et al., 2017; C. A. Murray & Shams, 2023; Thelen & Murray, 2013), but they go above and beyond these earlier findings by showing that these effects span retention intervals considerably longer than those used so far. Please note that all studies, except for Meyerhoff et al. (2016, 2022), have used retention intervals in the order of minutes. Meyerhoff et al.’s studies also found crossmodal matching effects over periods of 1 day and 1 week, but their matching versus mismatching conditions consisted of synchronized versus desynchronized movie clips, thus including multiple congruent/incongruent objects instead of single objects. Despite the fundamental principles might not be different, our study allows more direct extrapolation to most of the crossmodal memory studies in the literature, also using single object images and sounds (see the Introduction for a brief review).

Another finding to emerge from this first experiment is that crossmodal congruence impacted the recollection process, hinting at another of the traditional hallmarks of episodic memory effects. One recent study has also addressed the effects of crossmodal semantic congruence adopting the DPSD model (Duarte et al., 2023). In their Experiment 2, Duarte et al. (2023) used a protocol very similar to the one used here, except that the test phase occurred almost immediately after encoding (in their case, participants made a size judgment task about the visual object of the multisensory pair). They showed, quite convincingly, that the effect of crossmodal congruence was entirely due to a difference in recollection without an effect on familiarity. Despite both Duarte et al. and we anticipated (and found) a robust effect of crossmodal congruence on recollection, these two studies differ in the effects seen on familiarity. Since neither Duarte et al.’s nor our initial hypothesis hinged specifically on the familiarity outcome, we consider these two studies to agree in their core conclusions, and we can only speculate about the potential origin of this disparity. One important difference between studies is retention interval (several minutes vs. 2 days). Despite the large difference in retention interval, however, the values of Duarte’s (2023) DPSD parameter estimates and those in the present Experiment 1 were not so different on average, except the familiarity parameter for incongruent items, much lower in our case (for Duarte et al. and Experiment 1, respectively: R_cong_ = 0.26 [*SD* = 0.20] vs. 0.38 [*SD* = 0.20]; R_incong_ = 0.18 [*SD* = 0.18] vs. 0.17 [*SD* = 0.12]; F_cong_ = 1.54 [*SD* = 0.73] vs. 1.52 [*SD* = 0.53]; 1.44 [*SD* = 0.60] vs. F_incong_ = 1.0 [*SD* = 0.37]). One could make the interpretation that longer retention intervals especially degraded familiarity for items experienced with incongruent sounds. There are other differences between experiments: (a) that Duarte et al. included a control condition consisting of meaningless sounds and we did not; (b) we used a conditional inclusion rule for stimulus inclusion as a data quality control; and (c) the two experiments used slightly different encoding tasks. We return to this subject in the Discussion section.

## Experiment 2: Effect of crossmodal congruence in older adults

In Experiment 1 we have found that crossmodal semantic congruence during encoding of images facilitates later recognition of these images in episodic long-term memory. In Experiment 2, we aimed at extending this main result of crossmodal congruence investigating its prevalence at an older age (> 50 years old) and compare the crossmodal gain effect with the young adult sample tested in Experiment 1.

We hypothesized that in addition to any memory facilitation owing to the congruence effects of crossmodal inputs, subsequent memory may be overall decreased in the older population. The relevant question is, however, if the age-related memory decline would lead to an exacerbated crossmodal gain, given the increased potential for improvement. This pattern should be reflected in the following effects on memory performance (as measured with R, F, and *d′*): First, we expected to reproduce a robust crossmodal congruence advantage in this experiment, just like in Experiment 1. Second, we anticipate overall poorer performance in the older group, compared with the younger group tested in Experiment 1, at least in recollection (see Koen & Yonelinas, 2016). Finally, one could expect that the magnitude of the crossmodal gain might increase with diminishing effectiveness of the respective individual modalities, a phenomenon called inverse efficiency principle (e.g., Nidiffer et al., 2016). If memory decrements at older age substantiate a greater crossmodal congruence gain, we expect an exacerbated crossmodal gain in the older group, which should reflect in an interaction between crossmodal congruence and age.

### Methods and data analyses

#### Participants

As for Experiment 1, we used G*Power (Faul et al., 2007) to calculate the required sample size. Considering the correlation between *d′* for congruent and incongruent stimuli obtained in Experiment 1, *r* = 0.83, we estimated that we needed a sample size of at least 116 participants, to detect an interaction of congruence and age with a small effect size *f* of 0.10, with a power of 0.95, and an alpha level of 0.05. Because we already have datasets from 58 young adult participants in Experiment 1, for Experiment 2 we recruited an additional 58 older participants for a total sample size of *N* = 116 in the final analysis including both studies. We used the same criteria for inclusion of participants’ data and recruitment procedure with Prolific as in Experiment 1, except that we accepted participants between 50 and 75 years of age. In the final sample, mean age was 55.12, *SD* = 3.91, min = 50, max = 65; *SD* = 3.91 years. Before reaching the final sample of *N* = 58 (29 women) older adults, four participants were excluded for having too low a performance in the arithmetic distraction task, 13 were excluded for too low a performance in the memory task, and one was excluded for not reaching 70% agreement at encoding.

### Results

#### Encoding and distraction task

Across all items, in Experiment 2, the average interparticipant congruence rating was 2.29 (*SD* = 0.12) over 4. Participants gave a mean of 45.53% congruent responses (responses 3 or 4 for the congruence rating task, *SD* = 3.63%), and 54.47% incongruent responses (responses 1 or 2, *SD* = 3.69%). Participants gave on average 87.8% (*SD* = 6.0%) congruent responses to audiovisual pairings categorized as congruent by design, hence rendering on average 81.7 (min–max: 66–89) valid items for the congruent condition in the memory test. For pairs categorized as incongruent by design, participants gave on average 96.8% (SD = 3.3%) incongruent responses, rendering on average 90.0 (min–max: 75–93) valid items for the incongruent condition in the memory test (see Appendix 4 for distribution of responses). The mean reaction time for congruent responses was 1.90 s (*SD* = 0.41), and for incongruent responses was 1.75 s (*SD* = 0.44). The intraclass correlation coefficient measuring agreement between single fixed raters for the items in the congruence rating task was 0.82. The interparticipant average hit rate for the distraction task right after the encoding phase, where participants had to choose the larger of two numbers, was 0.88 (*SD* = 0.05).

#### Recognition task

##### DPSD model results

**Fig. 2:**
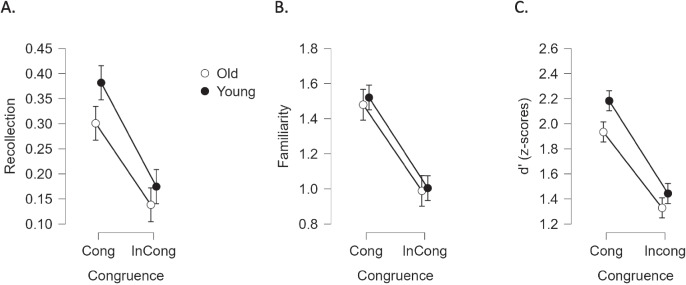
Average (± 95 CI) performance for (**A.**) recollection (**B.**) familiarity, and (**C.**) *d′* shown for congruent and incongruent conditions in each age group (young adults from Experiment 1, and older adults from Experiment 2; closed and open symbols, respectively)

Before applying the DPSD model, we had to apply the same criteria as in Experiment 1, to ensure an even distribution along all the confidence level responses, which required excluding seven of the older participants, leaving a sample size of *N* = 51 for the DPSD model analyses for Experiment 2. Mean recollection (R parameter) for congruent items was 0.30 (*SD* = 0.22) and 0.14 (*SD* = 0.14) for incongruent ones (Wilcoxon signed-rank test; *Z* = 5.59, *p* < 0.001). The data deviated from normality (*W* = 0.934, *p* = 0.007), and the difference was also significant with a two-tailed *t* test, *t*(50) = 6.87, *p* = 9.74e-9, Cohen’s* d* = 0.96, indicating a large effect size. Familiarity (F parameter) for items in the congruent condition was 1.50 (*SD* = 0.55), whereas F in the incongruent condition was 0.99 (*SD* = 0.34). F for congruent items was significantly greater than for incongruent ones (Wilcoxon signed-rank test; *Z* = 5.32, *p* < 0.001). The data were normally distributed (*W* = 0.970, *p* = 0.23), and the difference was also significant with a two-tailed *t* test, *t*(50) = 7.99, *p* = 1.74e-10; Cohen’s *d* = 1.12, indicating a large effect size. We then pooled together the datasets of Experiments 1 and 2 to run 2 × 2 analyses of variance (ANOVAs) for R and F, including age as a between-subjects factor (Fig. 2A–B). For recollection, the ANOVA returned significant effects of age, *F*_(1,107)_ = 3.99, *p* = . 048; η_p_^2^ = 0.036, and of congruence, *F*_(1,107)_ = 118.61, *p* < 0.001, η_p_^2^ = 0.526, but no interaction between the two, *F*_(1,107)_ = 1.73, *p* = . 191. As expected, the older age group had poorer recollection scores than the younger group (0.22 vs. 0.28). Despite congruent events being recollected more often than incongruent ones, the magnitude of this crossmodal congruence effect did not vary across age groups. Regarding familiarity, the ANOVA indicated that age group was not significant, *F*_(1,107)_ = 0.13, *p* = 0.719. As with recollection scores, however, the effect of congruence was significant in the expected direction *F*_(1,107)_ = 164.76, *p* < 0.001, η_p_^2^ = 0.60, but there was no interaction, *F*_(1,107)_ = 0.10, *p* = 0.75.

##### *d′* scores

The effect of congruence on *d′* (Fig. 2C), tested independently in the older age group, was significant (Wilcoxon signed-rank test; *Z* = 5.94, *p* < 0.001). The data deviated from normality (*W* = 0.945, *p* = 0.019), and the difference was also significant with a two-tailed *t* test, *t*(1,57) = 10.7; congruent *d′* = 1.93 [*SD* = 0.424] versus incongruent *d′* = 1.33 [*SD* = 0.309], Cohen’s *d* = 1.38, with a large effect size. As for the age by congruence ANOVA on *d′*, the test returned a significant main effect of congruence, *F*_(1,114)_ = 282.04, *p* = 1.31e10^−32^, η_p_^2^ = 0.395, and a main effect of age, *F*_(1,114)_ = 7.99, *p* = 6.00e10^−3^, η_p_^2^ = 0.070, with older people having lower *d′* scores. There was no significant interaction, *F*_(1,114)_ = 2.76, *p* = 0.10.

### Discussion

Experiment 2 replicated and extended, to an older age group, the crossmodal semantic congruence memory gain over long retention intervals observed in Experiment 1. This result grants further support to the hypothesis that crossmodal semantic congruence increases the elaboration or ‘spread’ of the encoding for the event (i.e., total breadth of analysis carried out), compared with semantically incongruent information, as discussed in Craik & Tulving (1975). Although older participants displayed lower memory performance (at least in terms of recollection and *d′*), the magnitude of the crossmodal congruence gain did not vary across age groups. This latter result answers, for now, one of the questions in this experiment: Will older participants capitalize more on crossmodal congruence, given their reduced memory capacity? It seems, from our result, that this is not the case. However, upon closer inspection, the evidence toward the null hypothesis (no difference) is anecdotal for recollection (BF01 = 2.27) and for *d′* (BF01 = 1.47), whereas for familiarity evidence for the null hypothesis is slightly more substantial (BF01 = 4.70).

Unfortunately, there is only one other study testing crossmodal semantic congruence effects in an older age group. Heikkilä et al. (2018), like our Experiment 2, compared a sample of older adults (70 years old on average) with a sample of younger adults (23 years old on average). Unlike us, Heikkilä et al.’s study found larger crossmodal gain for younger adults than older ones. Their experiment, however, tested memory for object sounds when presented with congruent object images, compared with single-modality object sounds; thus, a different combination from the one studied here. Also, their memory set was smaller (52 items), the encoding phase involve intentional study, and the test was delivered immediately after study. Considering these differences, it is difficult to compare their outcomes with ours.

It is perhaps worth noting two further results emerging from Experiment 2. First, the crossmodal congruence gain was observed not only in terms of recollection, as expected a priori and just as Duarte et al. (2023) reported for much shorter intervals, but also for the familiarity parameter in this new group of older participants. This confirms the outcomes of Experiment 1 and a possible fundamental difference between Duarte et al.’s result (crossmodal gain observed in recollection but not in familiarity) and ours (crossmodal gain observed in both parameters). Second, the overall decline in memory performance for the older age group could be principally attributed to worse recollection compared with the younger group, but this decline was not observed for familiarity (here, Bayesian tests rendered extremely strong evidence for the null difference in familiarity between age groups, BF01 > 100). This result replicates Koen and Yonelinas’s (2016) earlier findings, who also showed that age-related memory decline selectively affected recollection, not familiarity. This latter result bears no clear implications for the main question of the present study (crossmodal semantic congruence effects on memory), but it may help understand the nature of the age differences in episodic memory. It seems clear that age differences in overall memory performance in our protocol were more related to the quality of the episodic memory representation than to any differences in perceptual fluency. This may perhaps explain the differences with respect to Heikkilä et al.’s (2018), who claimed that their task, with much shorter presentations, may have led to worse perceptual encoding in the older participants.

## Experiment 3: Effects of distribution frequency (rarity)

In the two initial experiments of this study, subjects were presented with as many crossmodal semantically congruent events as incongruent ones during the encoding session. An approximately equivalent proportion of congruent and incongruent crossmodal events is also typical of previous experiments using congruence manipulations (Craik & Tulving, 1975; Heikkilä et al., 2015, 2017; Kim & Cabeza, 2007a, 2007b; Packard et al., 2017, 2020; van Kesteren et al., 2012), including many of the crossmodal studies discussed above (e.g., Heikkilä et al., 2015, 2017; Heikkilä & Tiippana, 2016). We reasoned, however, that a balanced ratio between congruent and incongruent events may not be necessarily representative of everyday life experience. On the contrary, most of the time, multisensory events provide correlated/congruent information across their modalities, a fact that has been related countless times in the introduction of multisensory review papers and books (for reviews, see Shams & Seitz, 2008; Stein, 2011). Hence, crossmodal incongruence must be exceptional in everyday experience, making these events rather unique and salient. This may be consequential for later memory and perhaps moderate the role of semantic congruence in everyday life conditions rather differently than what we see in the typical laboratory experiment. In short, encountering a barking cat on our way back from work will probably be far more memorable than passing by a meowing one. At least one previous study, Glicksohn et al. (2023) found better memory for crossmodally incongruent items than for congruent ones when they were rarer (12.5% vs. 82.5%). In Glicksohn et al.’s study, however, perceptual salience was conflated with event rarity, and it is difficult to tease apart the contribution of these two factors in the outcome.

Outside the domain of crossmodal studies, several memory phenomena attest to the importance of salience, surprisal, and higher memory for infrequent oddballs. Novelty has been noted as a likely candidate for an important determinant of later memory (Bunzeck & Düzel, 2006; Tulving & Kroll, 1995; van Kesteren et al., 2012). For example, infrequent items that are distinctive in that they deviate from the surrounding items they are presented with, are often better remembered (Gretz & Huff, 2020; Hunt, 1995, 2013; Siefke et al., 2019). Similarly, bizarre imagery has been shown to lead to increased memory for sentences (Mcdaniel & Einstein, 1986). Surreal, semantically conflicting imagery also has been shown to be better remembered (Ruzzoli et al., 2020). This appears to agree with predictive coding memory models (Henson & Gagnepain, 2010; Reichardt et al., 2020) which postulate that prediction error in response to perceptual, semantic or emotional oddballs activates learning-related brain mechanisms (Strange et al., 2000). In addition, according to previous studies using a DPSD approach, the improvement effect of novelty on memory is due mostly to familiarity, and not recollection, after incidental encoding (Kishiyama & Yonelinas, 2003).

Experiment 3 was designed to test the effect of distribution frequency of congruent and incongruent items. We collected data from two new experiments, one in which incongruent events were rare (approximately 1 in every 10; Experiment 3a) and one with the opposite distribution (approximately only 1 in every 10 events was congruent; Experiment 3b). These experiments were run consecutively as independent studies, with separate registrations. However, their predictions have been tested and reported below as a between-subjects design, pooling both datasets combined with the dataset of Experiment 1 (50%, even distribution). This has been done for brevity and to allow for a more complete and direct test of the frequency effects.

### Methods

#### Participants

For both Experiment 3a and 3b, we used G*Power (Faul et al., 2007) to calculate the required sample size, considering the correlation between *d′* for congruent and incongruent stimuli obtained in Experiment 1 of 0.83. We estimated that we needed a sample size of at least 56 participants per distribution condition, to detect an interaction (between congruence and distribution) with effect size *f* of 0.10, with a power of 0.95 and an alpha of 0.05. Because we already have 58 participants in Experiment 1, we recruited a new sample of *N* = 58 each for Experiments 3a and 3b. The age of participants was, on average, 24.58 years old (*SD* = 4.67; min = 18, max = 35) in Experiment 3a, and 25.74 years old (*SD* = 4.86, min = 18, max = 35) in Experiment 3b.

We used the same recruitment protocol, hourly compensation, and performance criteria for inclusion of participants’ data as in the previous experiments, except for the requirement of a minimum amount of intermediate confidence responses, given the small number of trials in some conditions. All other details of the procedure are the same as in Experiment 1. For Experiment 3a, before reaching the final sample of 58, four participants were excluded due to having too low a performance in the arithmetic distraction task, two were excluded due to having too low an accuracy during the encoding task, four were excluded for too low a performance in the memory task, and one was excluded due to being an extreme outlier *d′* in the incongruent condition (three times greater than the interquartile range). In Experiment 3b, before reaching the final sample of *N* = 58 (29 women), three participants were excluded for having too low a performance in the arithmetic distraction task, four were excluded for too low a performance in the memory task, and one was excluded for not logging any responses during the encoding task.

#### Stimuli and procedure

The methods were the same as for Experiment 1, except for the following. For Experiment 3a, from the total 372 images, 186 stimuli were used in the encoding protocol, of which 22 (11.83%) were assigned to the incongruent condition, and the remaining 164 (88.17%) were assigned to the congruent condition. For Experiment 3b, the distribution of congruence and incongruence was reversed, at the encoding stage. Hence, from the total of 372 images, 186 were included in the encoding protocol and from those, 22 (11.83%) were assigned to the congruent condition, and the remaining 164 (88.17%) were assigned to the incongruent condition. The rest of the procedure was otherwise carried out just as for Experiment 1.

We conducted simulations using empirical data from the previous experiment to estimate the effect size when using a reduced set size of 22 items (incongruent condition of Experiment 3a and congruent condition of Experiment 3b). Such small set size is in principle insufficient to calculate a reliable ROC curve (60 items per condition are recommended for stable ROC curves; Juola et al., 2019; Yonelinas, 2002), and therefore recollection and familiarity parameters as estimated by the dual-process signal detection (DPSD) model (Koen et al., 2017). However, the simulations showed that the effect of congruence on *d′* was of the same magnitude as with larger set sizes. Therefore, even in the less populated condition, the present experiment should provide a good ratio to generate a stable *d′* estimate, and the sensitivity of recognition memory for rare incongruent items. We therefore decided to use *d′* as our principle measure for hypothesis testing, and present R and F in exploratory analyses.

The incongruent items in Experiment 3a (and the congruent items in Experiment 3b) were selected randomly from those incongruent (congruent, respectively) audiovisual pairs in Experiment 1 that were rated with above average incongruence (congruence, respectively). In addition, sounds that did not seem incongruent enough were replaced by more obviously incongruent sounds. This selection was counterbalanced by applying two different random assignments across individuals. The hit rate was calculated for both congruent and incongruent conditions, and then *z* transformed to calculate *d′* scores. The distraction task was shortened to 50 trials, judged to be long enough for our purposes.

Shapiro–Wilk tests for each combination of factor levels showed that all data conformed to normality (*W* > 0.966, and all *p* values > 0.123).

#### Predictions

If the effect of salience by rare/odd events is sufficiently large, then we would expect that rarer items will be better remembered than common ones. This means that incongruent items should be remembered relatively more in Experiment 3a than in Experiment 1, and in Experiment 1 than in Experiment 3b. The opposite trend is expected for congruent items. Variations in memory for incongruent/congruent items because of their rarity should lead to a modulation of the crossmodal congruence effect by rarity; when incongruent events are rare, the overall advantage of crossmodal congruence might decrease or even revert (as in Glicksohn et al., 2023). We will test this with an ANOVA, combining the data from Experiments 1, 3a, and 3b. Here, we would expect an interaction between crossmodal semantic congruence and salience (rarity) of stimuli on the memory sensitivity for images, measured with *d′.* In Experiment 1 we presented 50% congruent stimuli and 50% incongruent stimuli. In Experiment 3 there will be 90% congruent stimuli and 10% incongruent stimuli. We will run post hoc *t* tests to decompose the interaction if observed.

### Results

#### Encoding and distraction task in Experiments 3a and 3b

##### Experiment 3a (rare incongruent items)

Across all items, the average interparticipant congruence rating was 3.20 (*SD* = 0.18) over 4. Participants gave a mean of 77.3% congruent responses (responses 3 or 4 for the congruence rating task, *SD* = 5.56%) and 21.6% incongruent responses (responses 1 or 2, *SD* = 5.41%). Participants gave on average 87.4% (*SD* = 6.1%) congruent responses to audiovisual pairings categorized as congruent by design, hence rendering on average 143.4 (min–max: 119–159) valid items for the congruent condition in the memory test. For pairs categorized as incongruent by design, participants gave on average 97.3% (*SD* = 3.9%) incongruent responses, rendering on average 21.4 (min–max: 19–22) valid items for the incongruent condition in the memory test. The mean reaction time for congruent responses was 2.07 s (*SD* = 0.39), and for incongruent responses was 2.22 s (*SD* = 0.38). The intraclass correlation coefficient measuring agreement between single fixed raters for the items in the congruence rating task was 0.67. The interparticipant average hit rate for the distraction task right after the encoding phase, where participants had to choose the larger of two numbers, was 0.89 (*SD* = 0.05).

##### Experiment 3b (frequent incongruent items)

Across all items, the average interparticipant congruence rating was 1.45 (*SD* = 0.12) over 4. Participants gave a mean of 13.58% congruent responses (responses 3 or 4 for the congruence rating task, *SD* = 2.79%), and 85.79% incongruent responses (responses 1 or 2, *SD* = 2.94%). Participants gave on average 97.0% (*SD* = 4.0%) congruent responses to audiovisual pairings categorized as congruent by design, hence rendering on average 21.3 (min–max: 19–22) valid items for the congruent condition in the memory test. For pairs categorized as incongruent by design, participants gave on average 97.0% (*SD* = 3.3%) incongruent responses, rendering on average 159.1 (min–max: 136–164) valid items for the incongruent condition in the memory test (see Appendix 4 for distribution of responses). The mean reaction time for congruent responses was 2.00 s (*SD* = 0.51), and for incongruent responses was 1.90 s (*SD* = 0.52). The intraclass correlation coefficient measuring agreement between single fixed raters for the items in the congruence rating task was 0.84. The interparticipant average hit rate for the distraction task right after the encoding phase, where participants had to choose the larger of two numbers, was 0.89 (*SD* = 0.05).

#### Mixed effects ANOVA with d′ scores from Experiments 1, 3a and 3b

**Fig. 3:**
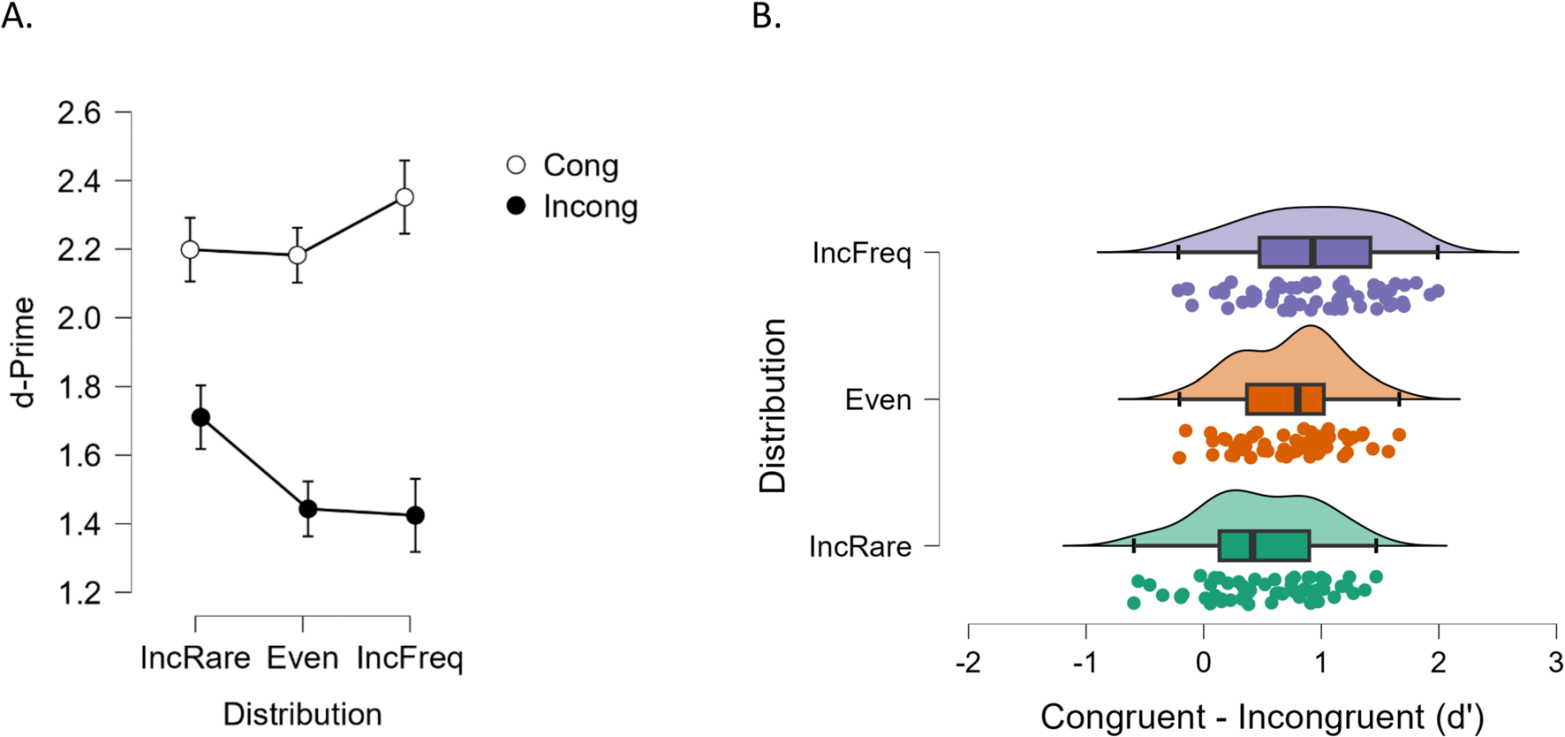
**A.** Average *d′* scores for the congruent and incongruent conditions in Experiments 3a (with rare incongruent events: IncRare), Experiment 1 (with equal distribution of incongruent and congruent events: Even), and Experiments 3b (with frequent incongruent events: IncFreq). **B.** Distribution of individual *d′* difference scores (congruence effect) for each distribution. (Colour figure online)

The ANOVA on *d′* included distribution frequency (rare incongruent items, even distribution, frequent incongruent items) as a between-subjects factor (datasets of Experiments 3a, 1, and 3b respectively; each *N* = 58), and congruence (congruent vs. incongruent) as a within-subjects factor (see Fig. 3A). The number of items per category logically varied from experiment to experiment. We opted to preserve all trials for analysis, at the risk of unequal variances, which were tested and compensated for analytically, when necessary. The ANOVA returned a significant main congruence effect, *F*_(1,171)_ = 352.11, *p* < 0.001, η_p_2 = 0.673, and a significant interaction between congruence and distribution, *F*_(2,171)_ = 11.06, *p* < 0.001, η_p_2 = 0.115. No main effect of distribution was detected in this test, *F*_(2,171)_ = 1.66, *p* = 0.193. To follow-up the significant interaction, we calculated the net congruence effect per distribution condition (*d′* congruent − *d′* incongruent) and submitted it to a new ANOVA with one between-subject factor (distribution; see Fig. 3B). The test returned a significant effect, *F*_(2,171)_ = 11.06, *p* < 0.001, η_p_2 = 0.115. Levene’s test was marginally significant (*p* = 0.051) indicating potential lack of homogeneity; however, the Brown–Forsythe correction returned equivalent outcomes, *F*_(2,162.43)_ = 11.06, *p* < 0.001, η_p_2 = 0.115. This mostly reflected that the effect of crossmodal semantic congruence was largest when incongruent items where frequent, and smallest (yet, positive and significant) when incongruent items were rare. Post hoc *t* tests (Bonferroni corrected) confirmed this patter statistically: Crossmodal congruence gain was significantly smaller in the incongruent rare distribution compared with the even distribution (*t* = − 2.68, *p* = 0.024, Cohen’s *d* = − 0.498, and compared with the incongruent frequent distribution (*t* = − 4.68, p < 0.001, Cohen’s* d* = − 0.870, whereas the numerical difference between even and incongruent frequent distribution did not reach significance (*t* = − 2.01, *p* = 0.139).

#### Mixed effects ANOVA with DPSD model parameters for Experiments 1, 3a, and 3b

Given the small number of trials in some of the conditions (specifically, incongruent trials in incongruent-rare Experiment 3a, and congruent trials in incongruent-frequent Experiment 3b), the outcomes of the DPSD model were highly variable in some cases. We detected five extreme outliers with *F* values more than three times greater than the interquartile range (*N* = 1 in the incongruent condition, *N* = 3 in the congruent condition, and *N* = 1 in both). These participants’ datasets were removed from the analyses of both R and F parameters. For the remaining individual datasets, model fit was on average *R*^2^ = 0.982 (*SD* = 0.027).

##### Recollection

**Fig. 4:**
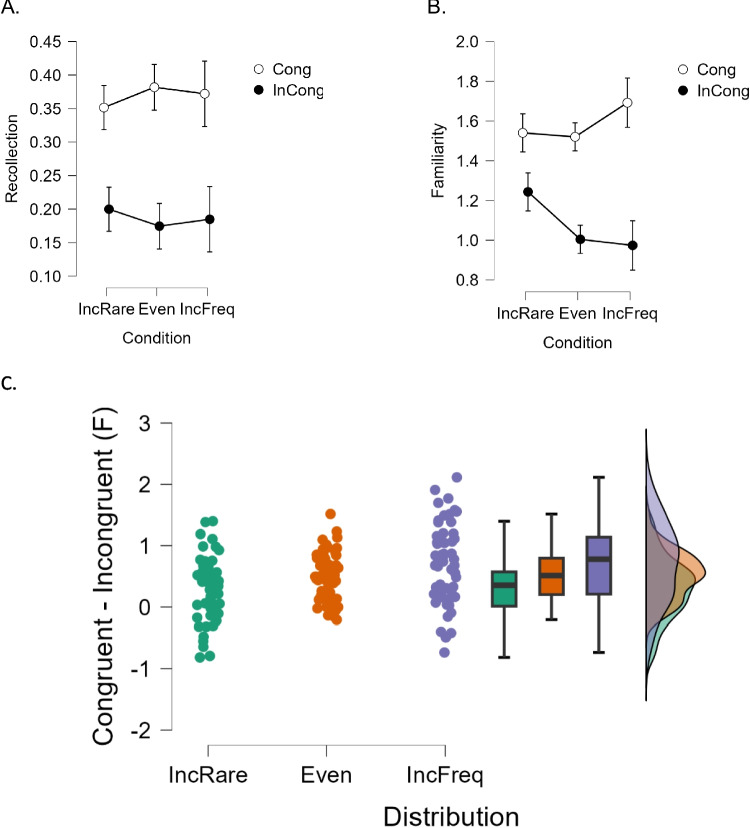
**A.** Average recollection scores across the different event distributions of Experiment 3a (incongruent rate), Experiment 1 (even), and Experiment 3b (incongruent frequent). **B.** Average familiarity scores across the same three distributions. **C.** Distribution of individual congruence effects in familiarity across the three distributions in Experiments 3a (green), 1 (orange), and 3b (purple). (Color figure online)

The ANOVA with the within-subject variable congruence and the between-subject variable distribution frequency, returned a main effect of congruence, *F*_(1,166)_ = 131.149, *p* < 0.001, η_p_2 = 0.441, confirming better recollection of congruent events across the board. However, neither the main effect of distribution nor the interaction between distribution and congruence reached significance in this test—respectively, *F*_(2,166)_ = 0.005, *p* < 0.995; and *F*_(2,166)_ = 1.052, *p* < 0.351 (Fig. 4A).

##### Familiarity

The ANOVA on F returned a main effect of congruence, *F*_(1,166)_ = 162.1, *p* < 0.001, η_p_2 = 0.494, confirming greater familiarity of congruent events across the board, but no main effect of distribution, *F*_(2,166)_ = 0.978, *p* < 0.378. At variance with recollection, however, in the familiarity analysis the interaction between distribution and congruence reached significance, *F*_(2,166)_ = 9.067, *p* < 0.001, η_p_2 = 0.098 (Fig. 4B). To better characterize this interaction, we calculated the net effect of congruence (congruent F minus incongruent F) and submitted it to an ANOVA with distribution as the only factor (Fig. 4C). The significant outcome, *F*_(2,138.7)_ = 8.947, *p* < 0.001, η_p_2 = 0.098, Brown–Forsythe corrected, indicated the following numerical pattern for crossmodal congruence gain: incongruent rare < even < incongruent frequent. The simple contrast between incongruent rare and incongruent frequent resulted statistically significant (*t* = − 4.26, *p* < 0.001, Cohen’s *d* = − 0.808). The congruence gain was marginally smaller in the incongruent rare distribution than in the even distribution (*t* = − 2.24, *p* = 0.079), whereas the congruence gain in the even distribution was not significantly different than in the incongruent frequent distribution (*t* = − 2.06, *p* = 0.123, all Bonferroni corrected).

### Discussion

Three conclusions emerge from the findings of the distribution manipulations across Experiments 1, 3a, and 3b. First, the effect of rarity by infrequency moderated the crossmodal congruence effect, so that the crossmodal congruence gain was smallest when incongruent items were rare, and therefore more memorable. Despite this effect of rarity, which was small, the beneficial effect of crossmodal semantic congruence was larger and relatively resilient to the large distributional imbalances between congruent and incongruent items introduced here. That is, crossmodal semantic congruence led to a significant memory advantage even when incongruent events were rare. Finally, the effects of crossmodal congruence and of rarity seem to impinge on different, or at least nonoverlapping aspects, of the memory process. That is, whereas crossmodal congruence affected both familiarity and recollection, the effect of distributional frequency on crossmodal congruence had an impact at the level of familiarity alone.

## General discussion

In our everyday life we experience multitude of events, many of them providing complementary or coherent information through more than one sensory system. Here, we set out to understand the processes whereby this semantic coherence across the sensory modalities modulates memory for these events. Despite there being ample evidence that events whose semantic information is congruent across sensory modalities are better remembered (for reviews, see Matusz et al., 2017; C. A. Murray & Shams, 2023; Soto-Faraco & Spence, 2025; Thelen & Murray, 2013) compared with semantically incongruent ones, the picture is still incomplete. For example, evidence for this crossmodal gain was lacking regarding incidental episodic memory, especially for retention periods longer than just a few minutes. Here, we used the DPSD framework to reveal that recollection for images is substantially better if originally experienced with their congruent sounds 2 days earlier, in an incidental encoding task. Participants were unaware of the subsequent memory task until the moment of the test.

The greater levels of recollection for images originally encoded with their semantically congruent sounds, compared with semantically incongruent ones, can be interpreted as a contextual effect. This hypothesis is grounded on previous evidence for a positive relationship between prior knowledge and the formation of new episodic memories, or the so-called congruence memory effect (Bein et al., 2015, 2020; Craik & Tulving, 1975; DeWitt et al., 2012; Kole & Healy, 2007; Packard et al., 2017; Poppenk et al., 2010; van Kesteren et al., 2012, 2014), and inspired by the beneficial context effects found with visual (Heikkilä et al., 2015; Heikkilä & Tiippana, 2016; Iordanescu et al., 2008; Kvasova et al., 2019; Mastroberardino et al., 2015; Matusz et al., 2015; Meyerhoff & Huff, 2016; Seitz et al., 2006; Thelen & Murray, 2013) and auditory (Heikkilä et al., 2017; Moran et al., 2013; Thelen et al., 2015) events in the past. Current hypotheses to explain this crossmodal memory advantage are compatible with this concept. For example, the multisensory memory reactivation account (Matusz et al., 2017; Thelen et al., 2012) propose that congruent crossmodal experiences form richer, more robust multisensory representations that contribute to retrieval upon reexperiencing one of the original, single-modality components. This is consistent with the general hypothesis of redintegration (proposed in the nineteenth century by E. J. Hamilton; see Hollingworth, 1920), although the multisensory memory reactivation account specifically proposes multisensory brain representations to be involved. We reasoned, according to this framework, that crossmodal semantically congruent stimuli would benefit from such positive context effects due to the formation of multisensory representations, and therefore benefit from increased subsequent episodic memory in general and more specifically, recollection, the critical process supporting episodic memory function.

The evidence that crossmodal memory gains bear the hallmarks of episodic memory effects (long retention interval, deep level of processing, incidental encoding) generally supports the multisensory memory reactivation account (see Matusz et al., 2017; Thelen et al., 2012, for reviews of this account). This interpretation contrasts, for example, with Dual-Coding accounts, which would not explain crossmodal improvements based on multisensory memory representations. In addition, recent interpretations of crossmodal memory gains have negated a multisensory account altogether, and pointed to methodological confounds that obscured effects of encoding specificity (Pecher & Zeelenberg, 2022). Although our findings cannot directly speak to encoding specificity effects, the interpretation in terms of richer multisensory memory detracts from this critique (we will discuss this further, below).

### Effects of familiarity

Although the main prediction of the DPSD regarding crossmodal congruence (and main focus in Experiments 1 and 2) was recollection, the results suggested overall a strong increase in familiarity for semantically congruent crossmodal stimuli as well. Though an increase in familiarity was not unexpected, it was not as strongly predicted from the DPSD framework. Familiarity is generally found to be faster than recollection, and the familiarity parameter (F) is a continuous index estimating general memory strength, thought to be accompanied by a feeling of acquaintance, and described by most authors as being supported by more automatic, less controlled processes than recollection (Yonelinas, 2002). Thus, familiarity is thought to be beneficial in that it allows individuals to quickly recognize items encountered before, even in the absence of the recollection of any associated specific information about the context the item was first encountered in or its concrete features (Yonelinas, 2002; Yonelinas et al., 2010). That semantically congruent crossmodal stimuli benefit from advantages in both recollection and familiarity, at least when retention intervals are long, is doubly advantageous, and reflects a more general memory benefit of crossmodal semantics. It may reflect a modulation of a more widely acting mechanism that promotes both memory processes or perhaps an early mechanism that triggers downstream parallel effects that modulate each memory process separately. In this sense, our results are at variance with the previous study of Duarte et al. (2023) who found that crossmodal semantic congruence increased recollection, but not familiarity. As we have already discussed above, the main take home message from both studies is the consistent improvement in recollection. It is less clear how to interpret the inconsistent results on familiarity since neither of these two studies had a strong prediction. One can only speculate about some methodological differences between these two studies. First, the substantially different retention intervals might explain this difference. For example, the sense of familiarity may initially remain at similar levels for both congruent and incongruent items but dissipate faster over time for incongruently encoded events compared with congruent ones. Second, Duarte et al. included a meaningless sound condition, as well as the congruent and incongruent sound conditions. It is unclear exactly how this change may have selectively affected familiarity of incongruent items, however. Third, whereas in our study a stimulus was considered congruent/incongruent only if also classified as such by the participant, Duarte et al. did not use this conditional approach. Given the high contingency between congruence by design and subjective congruence (see Appendix 4), we believe this may not be the basis for such large difference. Lastly, the encoding task was different between Duarte et al. (who asked participants to judge whether the objects would fit in a suitcase or not) and the present study (where participants judged crossmodal congruence directly). These different tasks might have led to differences in encoding, such as, for example, different encoding depth, and lead to the different outcomes.

### Effects of age

This study also aimed to investigate possible interactions between healthy aging and memory, and the crossmodal congruence effect of congruence. We found the expected decline in overall memory performance in the older group, as reflected in *d′*, but also reproduced a dissociation between recollection and familiarity, that had been previously reported by Koen and Yonelinas (2016). Like them, our data showed that recollection, but not familiarity, was differentially higher in the younger group. However, the main goal of the age manipulation in our study was to understand a potential interaction—that is, whether the crossmodal congruence gain was modulated by age. The hypothesis to be tested was that given the poorer overall memory at older age, the potential gain afforded by crossmodal memory processes might be relatively larger in that sample. Our results did not bear out this expectation. The beneficial effect of crossmodal semantic congruence in the older age group was significant, but not different from that in the younger sample. This result is at variance with the previous report by Heikkilä et al. (2018), the only one comparing crossmodal effects on memory between a sample of young adults and a sample of older adults. However, one must here consider the important differences between studies, in modality (memory for sounds vs. images), design (blocked vs. mixed designs), retention intervals (immediate vs. 2 days). Unfortunately, based on Bayesian factors, the strength of evidence was insufficient to consider this null outcome as a robust indication of lack of effect in our study, and therefore we cannot yet settle this question. What we can say, perhaps, is that given that the sample size was precalculated to detect effects of medium size, if an additional gain of crossmodal congruence at an older age were finally to exist, it would likely be a small one.

### Effects of rarity

This study also set out to test whether increasing the salience of incongruent items by decreasing their relative proportion during the encoding phase to 10%, would cause an increase in memory performance in the incongruent condition. The issue of distribution frequency between congruent and incongruent items has been rarely addressed but it might be important on at least two counts. First, because according to some authors it could be at the base of some mixed outcomes, or even confounds, in past studies (Pecher & Zeelenberg, 2022; Thelen et al., 2015). Second, because the effects of relative frequency are relevant for understanding crossmodal memory in real-life environments, where incongruent events are rare (as well as potentially odd). We pooled the three datasets to perform an overall analysis with crossmodal congruence and distribution of incongruent/congruent events (10:90, 50:50, 90:10, respectively). The main finding to emerge was clear: Despite rarity had a positive impact on memory performance, the positive effect of crossmodal congruence superseded that of rarity. That is, when incongruent items were rare, they were remembered more, thus attenuating the advantage of frequent crossmodally congruent items; but this improvement due to rarity was far from upturning the crossmodal congruence gain. The pattern of results observed therefore highlights the two parallel mechanisms that impinge on episodic memory: one that increases memory for semantically congruent crossmodal stimuli and another which increases memory for items based on their relative rarity. Thus, both increasing the crossmodal semantic congruence, and increasing the saliency of items by reducing their frequency during the encoding phase, cause increases in memory performance for the images of such items, up to 48 h later. This provides evidence for our hypothesis that increasing the rarity of items will cause these to be processed as more distinctive, which increases attention and leads to an increase in subsequent memory.

The conclusions above are based on the analyses of *d′*, given that because of the small number of trials in some of the less populated conditions, the estimation of the DSPSD parameters recollection and familiarity was deemed less reliable. However, when examining DPSP parameters in an exploratory fashion, a second interesting outcome emerged. Namely, that the effects of congruence distribution on crossmodal congruence gain reported in the main analyses, appeared to be mainly due to a modulation in familiarity, but not recollection. If confirmed, this dissociation can indicate that not only crossmodal semantic congruence has a stronger effect than distributional rarity, but also of a different nature. Whereas crossmodal congruence benefits seem to be based on a richer, more detailed representation of the episode, leading to high confidence recognition, the effect of rarity seems to be based on a more automatic process, leading to an unconscious sense of having seen that episode before.

Given that the rather extreme distributional manipulation used here had only moderate effects on crossmodal congruence gains, one should perhaps be cautious when attributing large variations in the outcome of crossmodal effects to small changes in the distribution of the proportion of congruent and incongruent items (Thelen et al., 2015), let alone acting as a systematic confounding factor (Pecher & Zeelenberg, 2022). Yet one earlier finding did find a more extreme memory improvement for rare crossmodally incongruent items, amidst frequent congruent ones (Glicksohn et al., 2023). However, we must note here that the incongruent items used in Glicksohn et al.’s (2023) study were not only rare but also made conspicuously odd and more perceptually salient (by using alerting sounds, and several repetitions of the item). Therefore, we believe, the comparison between is difficult.

### Limitations

Finally, despite this study had clear outcomes and generally straightforward interpretation, it is fair to consider its limitations. First, there was a relatively high prevalence of congruent items rated as incongruent by participants (see Appendix 4). This might have been caused by the sounds being ambiguous with respect to the object category, given the large set of stimuli used. Although these stimuli were not included in the final analysis, this may have caused imbalances in the number of items for the congruent and incongruent conditions. Second, in Experiment 2 we targeted the age range 50–75, but all (included) participants were 65 or under, thus not meeting the general criterion of older adults (according to the NIH). Third, although this study attempted to capture some aspects of how crossmodal memories are encoded under natural circumstances, it is evident that our stimuli were not natural in many respects. Most notably, the stimuli were static rather than dynamic, thus breaking the crossmodal synchrony that naturally occurring events (such as a barking dog) have in the real world. This latter limitation is extensible to most memory studies investigating crossmodal congruence and opens an important challenge for future studies.

### Conclusions

In conclusion, the present results show that images presented simultaneously with semantically congruent sounds in an incidental encoding task are recollected better and become more familiar than images presented with other sounds. The main finding of our study is that this crossmodal effect, often reported in the literature but over much shorter retention intervals of seconds or minutes, is extensive to episodic memories tested over a much longer intervals of 2 days and encoded under incidental circumstances. A second finding to emerge from this study is that rarity increases subsequent memory for infrequent stimuli, compared with more frequent stimuli. Despite this rarity effect modulates the size of the crossmodal congruence advantage, the underlying mechanisms may be different, given the different patterns regarding recollection and familiarity. Our results regarding potential differences in crossmodal congruence effects at older age are less conclusive, and did not reveal any sign of positive evidence.

In our view, this crossmodal memory advantage is consistent with a general framework describing a positive relationship between prior knowledge and the formation of new episodic memories, or so-called congruence memory effect (Bein et al., 2015, 2020; Craik & Tulving, 1975; DeWitt et al., 2012; Kole & Healy, 2007; Packard et al., 2017; Poppenk et al., 2010; van Kesteren et al., 2012, 2014). In the present study, this gain concerns a crossmodal relationship between context and the relevant episode and it bears the hallmarks of an episodic memory improvement. Therefore, we believe that the findings generally support a mechanism based on multisensory memory representations, such as the one proposed in the multisensory memory reactivation account (Matusz et al., 2017; Thelen et al., 2012). The formation of these multisensory representations during encoding has a large effect on the subsequent contents of episodic memories for crossmodal events, and the subjective experience of remembering, as humans navigate their real-life multisensory environments.

To increase the validity of this episodic memory test, the participants were not informed of a subsequent memory test (incidental memory task) and were only presented with each stimulus once. According to some authors (Pause et al., 2013), when participants expect a subsequent memory test, or are presented with a stimulus several times, this activates other cognitive processes that are not representative of episodic memory formation under ecological conditions. Our results in this sense, plausibly, are informative of how episodic memories for crossmodal stimuli are formed during people’s daily routines. This increased ecological validity suggests practical implications of our findings.

The successful storage and retrieval of information in long-term memory is one of the main ways we measure successful learning of academic and extra-academic materials. Especially given the relatively large effect size found in the present experiment, we propose that the understanding of how semantically congruent crossmodal stimuli can help people remember information, could be implemented in educational endeavours (see, e.g., Lidestam et al., 2014; van Kesteren et al., 2014), such as for teaching new sets of symbols or other visual information in schools or similar settings. In driver's education, road signs could perhaps be presented together with associated sounds, to facilitate the learning process, to give a specific example. We hope that increased understanding of these crossmodal effects, especially for incidental memories over long spans, and their mechanisms, will lead to improve effectiveness for such practical purposes.

## Supplementary Information

Below is the link to the electronic supplementary material.

Supplementary file1 (DOCX 1107 kb)

## Data Availability

Data and analysis scripts are available at OSF (https://osf.io/45m76/?view_only=58ea77719ade453aafd6547126c05350) for peer review purposes. They will be made public if/when the paper is accepted for publication.

## Footnotes

1 Each Appendix cited in the text is available in the online version of the article as supplementary material.

## Code availability

Data and analysis scripts are available at OSF (https://osf.io/45m76/?view_only=58ea77719ade453aafd6547126c05350) for peer review purposes. They will be made public if/when the paper is accepted for publication.
