# Supplementary material for: Crossmodal semantic congruence and rarity improve episodic memory
Source: Mem Cognit. 2025 Feb 19;53(5):1396–418. doi: 10.3758/s13421-024-01659-9 (PMC12307457; doi:10.3758/s13421-024-01659-9)
Supplement: Supplementary file 1 — Supplementary file1 (DOCX 1107 kb) [file 13421_2024_1659_MOESM1_ESM.docx]

**Appendix**

Crossmodal Semantic Congruence and Rarity Improve Episodic Memory

**Appendix 1. Pilot Experiment**

Prior to data collection of the final version of Experiment 1 and the final formulation of the hypothesis, we ran a pilot experiment with N=15 to test the protocol and stimuli and see if they produced acceptable performance rates in the different tasks. The methods are as described above, except that for the pilot data we did not implement any minimum performance criteria for the inclusion of participants. In addition, in the pilot experiment there were 10 additional image-sound pairs which were subsequently removed, as well as 6 sounds used in the pilot experiment which were subsequently modified to make the stimuli less ambiguous. Furthermore, the incongruent sound-image pairs used in the pilot experiment were not always of different semantic categories, and the assignment of stimuli to the congruent and incongruent conditions was not counterbalanced across all participants. Concerning the image quality, all images were presented on the screen with the same size, but the image quality and resolution varied in some cases. As for the participants, the range of age included older participants as well (28-79 years of age). The recognition test was run one (N=7) or two (N=8) days after.

On average, participants rated 85.93% of the items we designed as congruent audiovisual pairs with a rating of 3 or 4, and they rated 89.73% of the items we designed as incongruent pairs with a rating of 1 or 2. We measured performance for audio-visually congruent and incongruent pairs with two parameters: d’ as typically calculated in standard one parameter memory models [z(hit rate) – z(fa rate)], and the recollection parameter R as calculated in the DPSD model (Yonelinas et al., 2010). The calculation and meaning of these measures have been explained above. Overall, in the memory test, average d’ was 1.78, with an average hit rate of 0.65 for the old images, and an average False-Alarm rate 0.11 for the new images. A Wilcoxon Signed-Ranks Test found statistically higher d’ memory (z(Hits)-z(False-Alarms)) (W = 119; p< 0.001) and recollection (R parameter, see Andrew P. Yonelinas et al., 2010) (W = 101; p< 0.001) for images that had been previously seen with semantically congruent sounds, compared to images shown with incongruent sounds. The effect size of the difference was medium to large for both d’ (hedges’ *g* = 0.95, 95% CI [0.59, 1.72]) and recollection (hedges’ *g* = 0.69, 95% CI [0.39, 1.31]). The encoding of the images (paired with the semantically congruent/incongruent sounds) was incidental, as participants were just instructed to rate how well sounds and images matched at encoding. These pilot results lead us to expect an increase for recollection I and d’ (z(Hits)-z(False-Alarms)) measures of memory for semantically congruent multimodal stimuli. We do not report a separate confidence analysis here as both d’ and recollection measures reported above already consider the responses given at different criteria thresholds, with recollection especially being associated to high-confidence responses.

**Appendix 2. d’ as a function of congruence rating**

To further explore differences in memory for items along the congruence rating scale, we compared d’ values as a function of congruence ratings. We organized trials in three bins from high incongruent (rating of 1), intermediate (ratings of 2 and 3), and high congruent (rating of 4). The pooling of ratings 2 and 3 was done due to the lower amount of trials with intermediate ratings. Below, we provide a table with descriptive statistics, and figures with the distribution of d’ scores per experiment. Single factor repeated-measures ANOVAs revealed a significant effect of congruence level for each experiment separately (all p<.001), and for the whole dataset pooled together (p<.001).

**Table A2**. Descriptive statistics for d’ scores for each experiment and congruence level used in the main analyses.

| **Experiment** | **Congruence Level** | **N** | **Missing** | **Mean d’** | **Std. Dev.** | **Min.** | **Max.** |
| --- | --- | --- | --- | --- | --- | --- | --- |
|  | C1 | 58 | 0 | 1.416 | 0.33 | 0.824 | 2.364 |
| Experiment 1 | C2-3 | 58 | 0 | 1.841 | 0.512 | 0.754 | 3.091 |
|  | C4 | 58 | 0 | 2.278 | 0.553 | 1.29 | 3.751 |
|  | C1 | 58 | 0 | 1.266 | 0.29 | 0.55 | 2.185 |
| Experiment 2 | C2-3 | 58 | 0 | 1.586 | 0.442 | 0 | 2.342 |
|  | C4 | 58 | 0 | 2.095 | 0.466 | 1.105 | 3.376 |
|  | C1 | 58 | 0 | 1.634 | 0.418 | 0.765 | 2.648 |
| Experiment 3a | C2-3 | 58 | 0 | 1.974 | 0.465 | 0.93 | 2.799 |
|  | C4 | 58 | 0 | 2.281 | 0.52 | 1.222 | 3.888 |
|  | C1 | 58 | 0 | 1.414 | 0.334 | 0.848 | 2.285 |
| Experiment 3b | C2-3 | 57 | 1 | 1.547 | 0.622 | 0 | 2.736 |
|  | C4 | 58 | 0 | 2.375 | 0.57 | 1.409 | 3.787 |

| A. Experiment 1  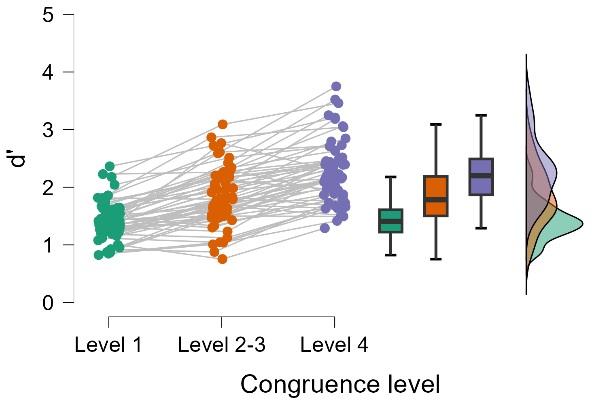 | B. Experiment 2  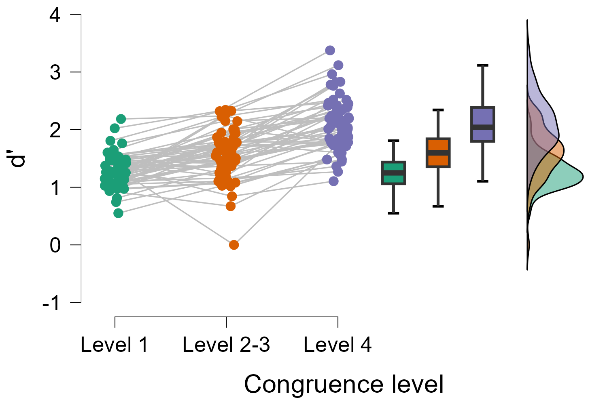 |
| --- | --- |
| C. Experiment 3a  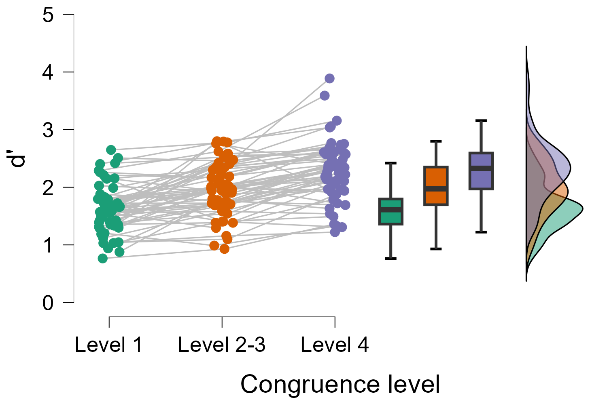 | D. Experiment 3b  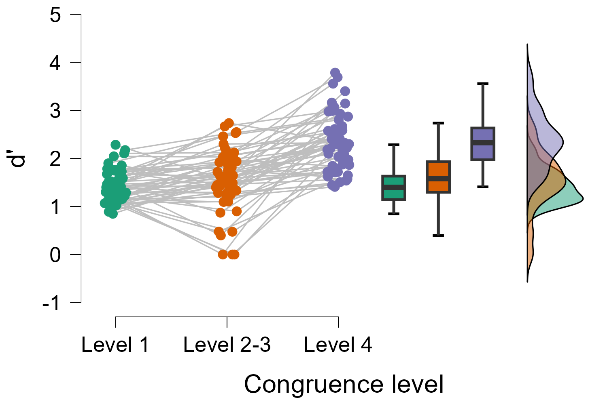 |
| **Figure A2.** Distribution of d’ scores as a function of congruence rating in A. Experiment 1, B. Experiment 2, C. Experiment 3a, and D. Experiment 3b. | |

**Appendix 3. Analysis by semantic category**

Semantic categories and high-level properties of images have been shown to determine to a certain extent their memorability (Isola, Parikh, Torralba, and Oliva 2011), with corresponding differences in subsequent recollection or familiarity (Broers and Busch, 2021). For example, faces have been shown to be more memorable than landscapes, and even more so if the faces seem kind, atypical, or trustworthy (Bainbridge, Isola, and Oliva, 2013). Although our stimulus set did not contain faces, we reasoned that similar phenomena may occur with other categories. To uncover any possible variations in the semantic congruence effect in the different semantic categories of stimuli included in our test, we ran a 4 by 2 Repeated Measures ANOVA on d’ with semantic category (4 levels: musical instruments, animals, transportation, and other objects) and crossmodal congruence (2 levels: congruent with a rating of 3 or 4, and incongruent with a rating of 1 or 2) as within-subjects factors, in the pooled dataset of all four experiments (N=231, 1 missing), using Greenhouse-Geisser correction for violations of sphericity. There was a main effect of semantic category (F(2.35, 542.0)=27.10; p<.001; η_p_^2^ =0.105), and a main effect of congruence in the same direction as in the main analyses (F(1,230)=507.2; p<.001; η_p_^2^ =0.688). The interaction between semantic category and crossmodal congruence was significant (F(2.30,528.46)=9.15; p<.001; η_p_^2^ =0.038) comparisons (adjusted alpha=0.01). We tested the simple main effect of congruence in each semantic category separately and found the effect of congruence was significant in all cases (all p<.001), in the same direction as in the main analysis (congruent > incongruent), with variations amongst categories (see Figure AX).

| A. Musical instruments  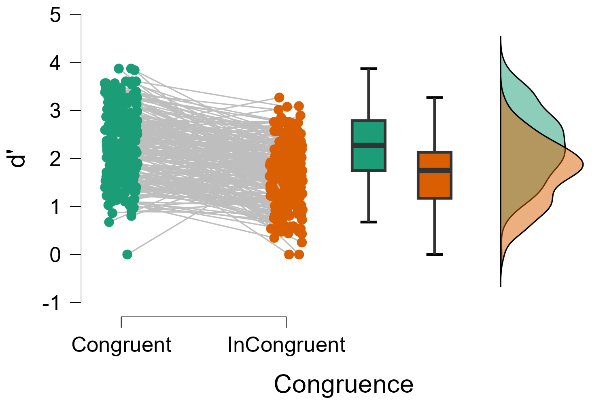 | B. Object  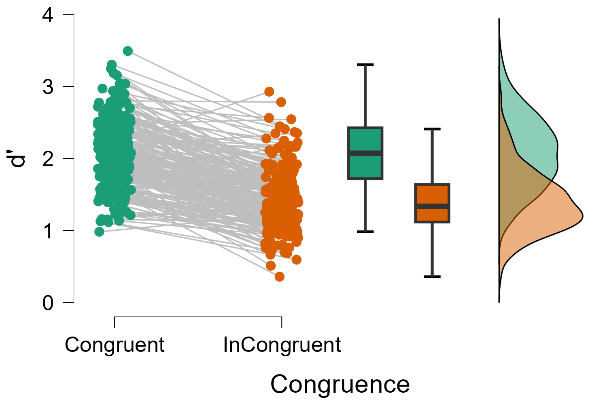 |
| --- | --- |
| C. Animal  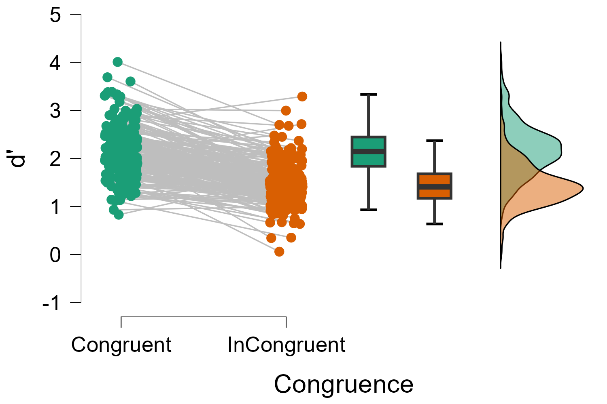 | D. Transport  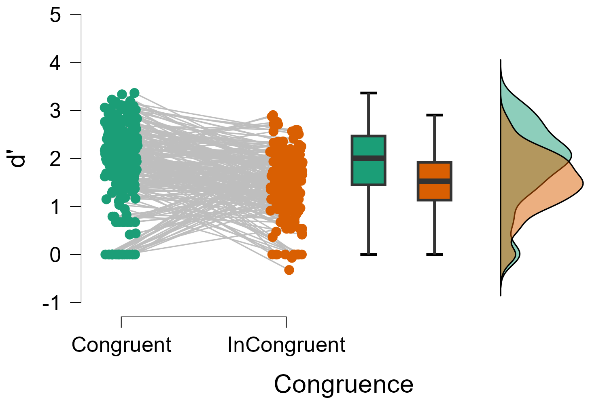 |
| **Figure A3.1.** Individual distribution of d’ scores for each subject, split by semantic category: A. Musical, B. Object, C. Animal, D. Transport. Data from all experiments pooled. | |

When looking at each experiment individually, the ANOVA for Experiment 1 returned main effects of Congruence (F(1,57)=362.42, p<.001, η_p_^2^ =0.864) and Category (F(2.42, 138.08)=11.99, p<.001, η_p_^2^ =0.174), but no interaction (p=.341). In Experiment 2 (older adults), the ANOVA revealed a similar pattern with a main effect of congruence (F(1,57)=297.73, p<.001, η_p_^2^ =0.839) and category (F(1.91, 112.02)=14.49, p<.001, η_p_^2^ =0.202), but no interaction (p=.198). In Experiments 3a and 3b, given the very low number of trials in the congruent and incongruent conditions within each category, the d’ estimates were therefore less reliable. In Experiment 3a, the ANOVA returned a main effect of congruence (F(1,56)= 239.03, p<.001, η_p_^2^ =0.810) and category (F(2.06, 145.91)=13.32, p<.001, η_p_^2^ =0.192), and a significant interaction (F(2.51, 140.75)=20.88, p<.001, η_p_^2^ =0.272). Simple main effects tested congruence for each semantic category separately revealed that congruence was significant in the expected direction for each semantic category (all, p<.001). In Experiment 3b, the ANOVA returned a main effect of congruence (F(1,57)=24.28, p<.001, η_p_^2^ =0.299) and category (F(1.93, 109.86)=27.37, p<.001, η_p_^2^ =0.324), and a significant interaction (F(2.08, 118.61)=65.51, p<.001, η_p_^2^ =0.535). Simple main effects tested congruence for each semantic category separately revealed that congruence was not significant for musical instruments, it was significant in the expected direction for objects (p<.001) and animals (p<.001), and with the congruent condition lower than the incongruent condition for transport (p<.001).

| A. Experiment 1  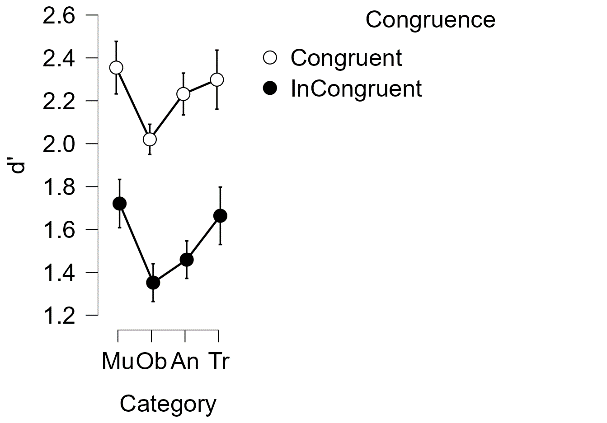 | B. Experiment 2 (Older adults)  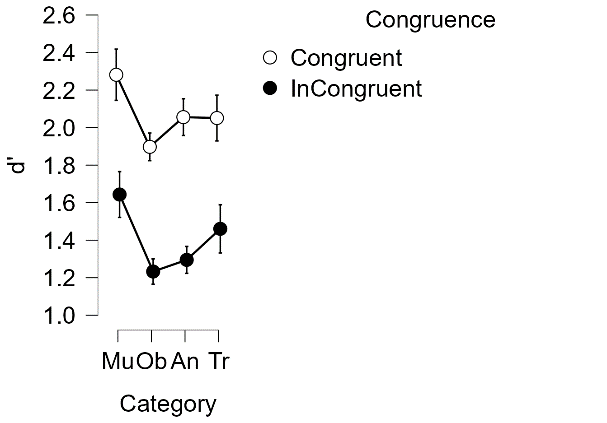 |
| --- | --- |
| C. Experiment 3a (Incongruent rare)  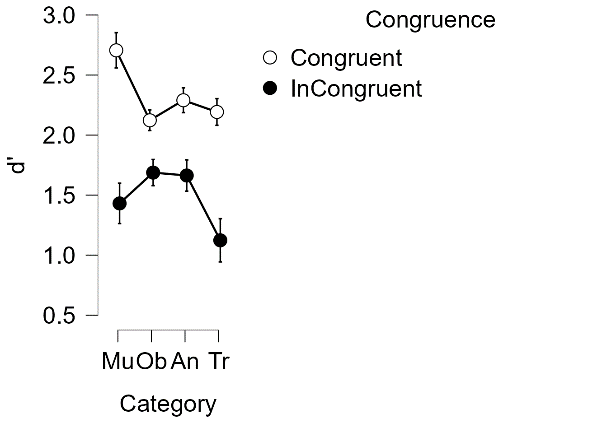 | D. Experiment 3b (Incongruent frequent)  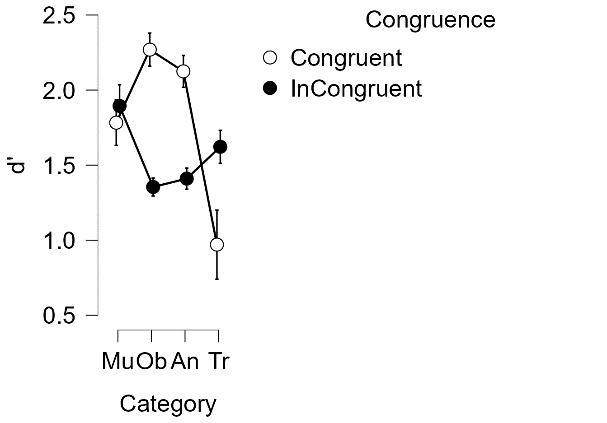 |
| **Figure A3.2**. Average d’ for congruent (open symbols) and incongruent (filled symbols) trials, as a function of semantic category (x-axis, Mu=Musical instruments, Ob=Objects, An=Animals, Tr=Transport), displayed separately for each experiment (A. Experiment 1, B. Experiment 2, C. Experiment 3a, D. Experiment 3b). | |

**Appendix 4: Encoding classification: descriptive statistics**

In this study, congruent trials in included in the analyses were those that had been included in the study as congruent by design, and later classified as congruent by the participant in the encoding phase (that is, assigned a congruence rating of 3 or 4, out of 4). The same procedure was followed for the incongruent trial category. Here, we show the distribution of events classified as congruent from the congruent category, and the events classifies as incongruent from the incongruent category for each experiment.

**Table A4**. Descriptive statistics for number of trials for each experiment and response category (CongR = Congruent response for congruent trials; IncongR = Incongruent response for incongruent trials). P(Cong|Cong) is the probability of a congruent response to a congruent trial. P(Incong|Incong) is the probability of an incongruent response to an incongruent trial. IQR = Interquartile range.

|  |  | | **Valid** | | **Median** | | **Mean** | | **Std. Dev.** | **IQR** | | **Min.** | | | **Max.** | |
| --- | --- | --- | --- | --- | --- | --- | --- | --- | --- | --- | --- | --- | --- | --- | --- | --- |
| CongR |  | Experiment1 |  | 58 | 79.500 |  | 78.724 |  | 6.725 |  | 7.750 |  | 60.000 |  | 91.000 |  |
|  |  | Experiment2 |  | 58 | 83.000 |  | 81.690 |  | 5.592 |  | 7.750 |  | 66.000 |  | 89.000 |  |
|  |  | Experiment3a |  | 58 | 144.000 |  | 143.397 |  | 10.085 |  | 16.000 |  | 119.000 |  | 159.000 |  |
|  |  | Experiment3b |  | 58 | 22.000 |  | 21.345 |  | 0.965 |  | 1.000 |  | 19.000 |  | 22.000 |  |
| IncongR |  | Experiment1 |  | 58 | 91.000 |  | 90.103 |  | 3.851 |  | 3.750 |  | 72.000 |  | 93.000 |  |
|  |  | Experiment2 |  | 58 | 91.000 |  | 89.983 |  | 3.035 |  | 3.000 |  | 75.000 |  | 93.000 |  |
|  |  | Experiment3a |  | 58 | 22.000 |  | 21.414 |  | 0.859 |  | 1.000 |  | 19.000 |  | 22.000 |  |
|  |  | Experiment3b |  | 58 | 161.000 |  | 159.086 |  | 5.381 |  | 6.000 |  | 136.000 |  | 164.000 |  |
| P(Cong\|Cong) |  | Experiment1 |  | 58 | 0.855 |  | 0.846 |  | 0.072 |  | 0.083 |  | 0.645 |  | 0.978 |  |
|  |  | Experiment2 |  | 58 | 0.892 |  | 0.878 |  | 0.060 |  | 0.083 |  | 0.710 |  | 0.957 |  |
|  |  | Experiment3a |  | 58 | 0.878 |  | 0.874 |  | 0.061 |  | 0.098 |  | 0.726 |  | 0.970 |  |
|  |  | Experiment3b |  | 58 | 1.000 |  | 0.970 |  | 0.044 |  | 0.045 |  | 0.864 |  | 1.000 |  |
| P(Incg\|Incog) |  | Experiment1 |  | 58 | 0.978 |  | 0.969 |  | 0.041 |  | 0.040 |  | 0.774 |  | 1.000 |  |
|  |  | Experiment2 |  | 58 | 0.978 |  | 0.968 |  | 0.033 |  | 0.032 |  | 0.806 |  | 1.000 |  |
|  |  | Experiment3a |  | 58 | 1.000 |  | 0.973 |  | 0.039 |  | 0.045 |  | 0.864 |  | 1.000 |  |
|  |  | Experiment3b |  | 58 | 0.982 |  | 0.970 |  | 0.033 |  | 0.037 |  | 0.829 |  | 1.000 |  |

|  | **Congruent responses to congruent events** | **Incongruent responses to incongruent events** |
| --- | --- | --- |
| **Experiment 1** | 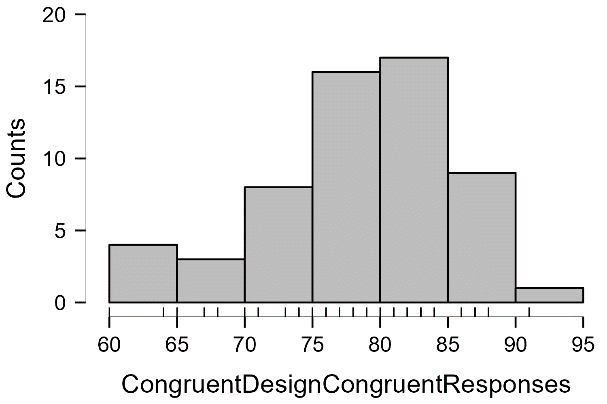 | 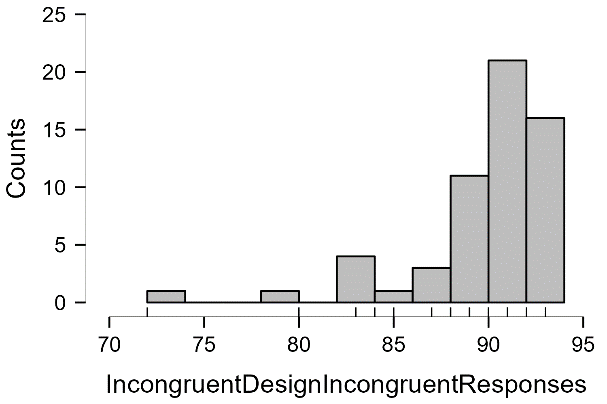 |
| **Experiment 2** | 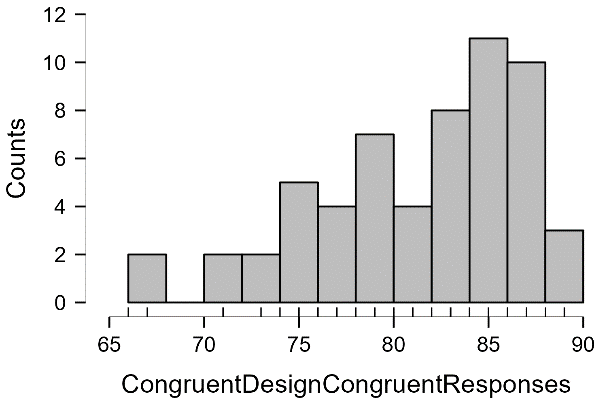 | 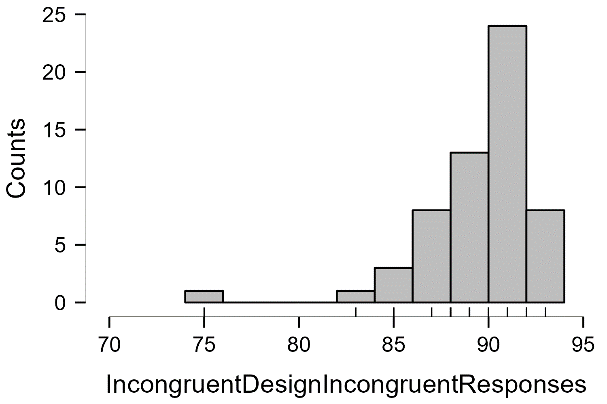 |
| **Experiment 3a** | 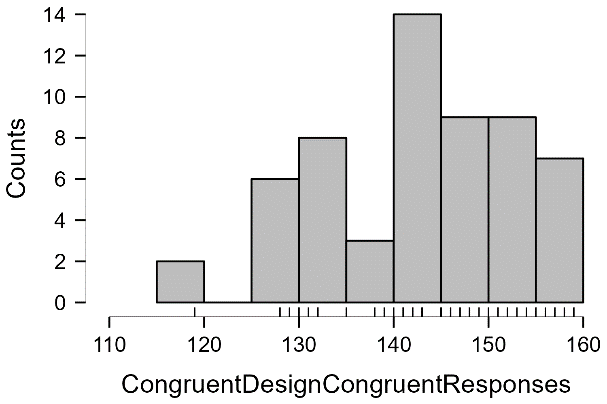 | 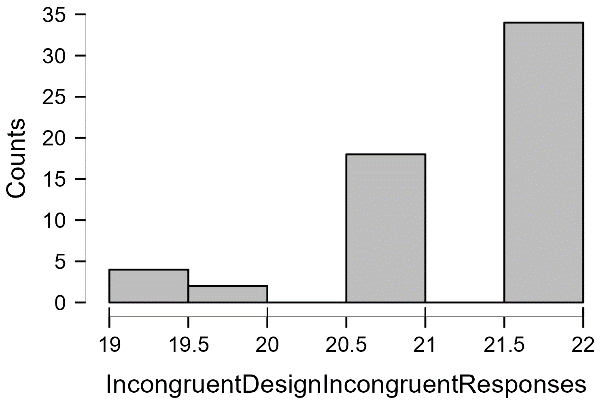 |
| **Experiment 3b** | 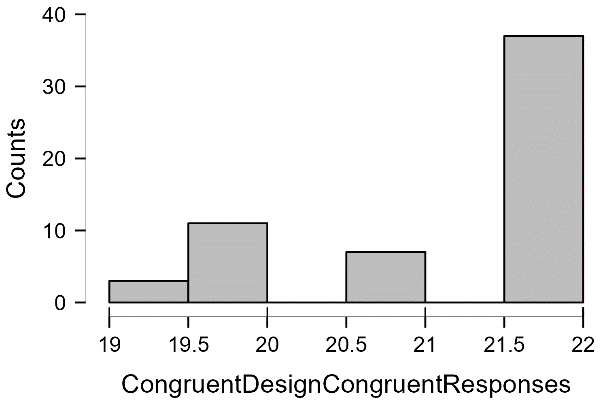 | 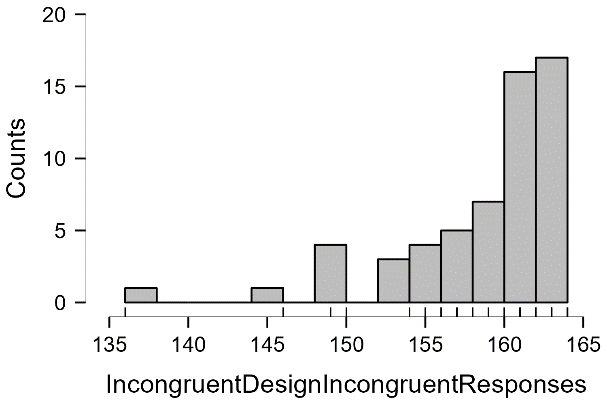 |

**Figure A4**. Distribution of number of trials per subject in each experiment and condition, included in the main analyses.

**Appendix 5: Dual-Process Signal Detection Model**

We defined the parameters as in the most common implementation of the DPSD. According to this framework, recognition performance for old and new items (subindices *o* and *n*, respectively) can be modelled as a function of Recollection I and Familiarity (F), as follows:

P(‘old’|old) = R+ (1 – R) F_o_

P(‘old’|new) = F_n_

Familiarity (F) is defined as a function Ф of *d’*, that is, the distance between the means of the old and new item distributions (Macmillan & Creelman, 2005), and the response criterion *c*. The Ф is the Gaussian cumulative distribution function which determines the portion of the normal distribution to the left of the z-score (Stanislaw & Todorov, 1999). Here it represents the cumulative normal proportion predicting the responses for old and new items based on familiarity:

F_o_= Ф(d’/2-c)

F_n_= Ф(-d’/2-c)

In our task, for the calculation of the signal detection theory parameters *d’* and *c* for the modelling of F we defined hits as an “old” response given to an old (target) item that appeared during the encoding phase, and false alarm as an “old” response given to a new (lure) item that had not appeared during the encoding phase. The confidence ratings correspond to the different rating bins which are cumulatively included in the normal proportion according to the shifting response criterion c. That is, the confidence levels or rating bins are required for ROC analysis and plotting across cumulative confidence intervals. Recognition results were analyzed with Matlab using the ROC toolbox (Koen et al., 2017). The formulas for the DPSD model (Koen et al., 2014) for the proportion of responses for each rating bin (𝐵𝑖) are:

P(𝐵𝑖|old) = 𝑅+ (1−𝑅) Ф(𝑐𝑘−𝐹,v𝐹)

P(𝐵𝑖|new) = Ф(𝑐𝑘,1)

Recollection I only influences the responses for old items (the target distribution), it is estimated as a probability, constrained to a value between 0 and 1 and has a starting value of 0.2. Familiarity (F) is estimated in d’ units like the d’ parameter of the UVSD model, with a starting value of 1 and limits of ±∞. V𝐹 is the variance of the familiarity strength distribution for target items and is estimated in standard deviation units. In this experiment, v𝐹 was constrained to 1. 𝑐1, 𝑐2, ..., 𝑐𝑘 are the criterion placement parameters of the model, determined by the number of bins minus 1, in this experiment with 6 bins there were 5 criterion placement parameters. The criterion locations are estimated as the interval between criteria, starting with the most conservative criteria. 𝐵𝑖 represents the 𝑖𝑡ℎ rating bin*,* P(𝐵𝑖|target) is the proportion of responses in each bin for old (target) items, and P(𝐵𝑖|lure) is the proportion of responses in each bin for new (lure) items. For more details about the DPSD model, ROC analysis and the ROC toolbox, please see Koen et al. (Koen et al., 2014), Yonelinas and Parks (Yonelinas & Parks, 2007), Yonelinas (Yonelinas, 1994), and Macmillan and Creelman (Macmillan & Creelman, 2005).

**Appendix 6: Group-wise ROC curves and model fit parameters for Experiments 1-3**

We present the group-wise average model fit summaries generated by the ROC Toolbox (Koen et al., 2017) for the DPSD model used to estimate the R and F parameters in the results presented in the main text.

**Figure A6.1** Experiment 1

**Figure A6.2** Experiment 2

**Figure A6.3** Experiment 3a

**Figure A6.4** Experiment 3b

**References**

Bainbridge, W. A., Isola, P., & Oliva, A. (2013). The intrinsic memorability of face photographs. *Journal of Experimental Psychology: General*, *142*(4), 1323. <https://doi.org/10.1037/a0033872>

Broers, N., Busch, N. The effect of intrinsic image memorability on recollection and familiarity. *Mem Cogn* **49**, 998–1018 (2021). <https://doi.org/10.3758/s13421-020-01105-6>

Isola, P., Parikh, D., Torralba, A., & Oliva, A. (2011). Understanding the intrinsic memorability of images. *Advances in neural information processing systems*, *24*. <https://proceedings.neurips.cc/paper/2011/hash/286674e3082feb7e5afb92777e48821f-Abstract.html>

Koen, J. D., Barrett, F. S., Harlow, I. M., & Yonelinas, A. P. (2014). *ROC Toolbox Manual Version 1.1.1*.

Koen, J. D., Barrett, F. S., Harlow, I. M., & Yonelinas, A. P. (2017). The ROC Toolbox: A toolbox for analyzing receiver-operating characteristics derived from confidence ratings. *Behavior Research Methods*, *49*(4), 1399–1406. https://doi.org/10.3758/s13428-016-0796-z

Macmillan, N. a N. A., & Creelman, C. D. (2005). Detection Theory: A User’s Guide. In *Detection theory: A user’s guide (2nd ed.).*

Stanislaw, H., & Todorov, N. (1999). Calculation of signal detection theory measures. *Behavior Research Methods, Instruments, and Computers*, *31*(1), 137–149. <https://doi.org/10.3758/BF03207704>

Yonelinas, A. P. (1994). Receiver-Operating Characteristics in Recognition Memory: Evidence for a Dual-Process Model. *Journal of Experimental Psychology: Learning, Memory, and Cognition*, *20*(6), 1341–1354. <https://doi.org/10.1037/0278-7393.20.6.1341>

Yonelinas, A. P., & Parks, C. M. (2007). Receiver Operating Characteristics (ROCs) in Recognition Memory: A Review. *Psychological Bulletin*, *133*(5), 800–832. <https://doi.org/10.1037/0033-2909.133.5.800>
